# High-throughput identification of repurposable neuroactive drugs with potent anti-glioblastoma activity

**DOI:** 10.1038/s41591-024-03224-y

**Published:** 2024-09-20

**Authors:** Sohyon Lee, Tobias Weiss, Marcel Bühler, Julien Mena, Zuzanna Lottenbach, Rebekka Wegmann, Miaomiao Sun, Michel Bihl, Bartłomiej Augustynek, Sven P. Baumann, Sandra Goetze, Audrey van Drogen, Patrick G. A. Pedrioli, David Penton, Yasmin Festl, Alicia Buck, Daniel Kirschenbaum, Anna M. Zeitlberger, Marian C. Neidert, Flavio Vasella, Elisabeth J. Rushing, Bernd Wollscheid, Matthias A. Hediger, Michael Weller, Berend Snijder

**Affiliations:** 1 Department of Biology, Institute of Molecular Systems Biology, ETH Zurich, Zurich, Switzerland; 2 Department of Neurology, Clinical Neuroscience Center, University Hospital Zurich and University of Zurich, Zurich, Switzerland; 3 Institute of Pathology, University Hospital Zurich, Zurich, Switzerland; 4 Membrane Transport Discovery Lab, Department of Nephrology and Hypertension and Department of Biomedical Research, Inselspital, University of Bern, Bern, Switzerland; 5 Institute of Biochemistry and Molecular Medicine, University of Bern, Bern, Switzerland; 6 Department of Health Sciences and Technology, Institute of Translational Medicine (ITM), ETH Zurich, Zurich, Switzerland; 7 Swiss Institute of Bioinformatics, Lausanne, Switzerland; 8 ETH PHRT Swiss Multi-Omics Center (SMOC), Zurich, Switzerland; 9 Electrophysiology Facility, University of Zurich, Zurich, Switzerland; 10 Department of Neuropathology, University Hospital Zurich and University of Zurich, Zurich, Switzerland; 11 Department of Neurosurgery, Cantonal Hospital St. Gallen, St. Gallen, Switzerland; 12 Department of Neurosurgery, Clinical Neuroscience Center, University Hospital and University of Zurich, Zurich, Switzerland; 13 Comprehensive Cancer Center Zurich, University Hospital Zurich, Zurich, Switzerland

**Keywords:** Translational research, CNS cancer, Phenotypic screening, Machine learning, Single-cell imaging

## Abstract

Glioblastoma, the most aggressive primary brain cancer, has a dismal prognosis, yet systemic treatment is limited to DNA-alkylating chemotherapies. New therapeutic strategies may emerge from exploring neurodevelopmental and neurophysiological vulnerabilities of glioblastoma. To this end, we systematically screened repurposable neuroactive drugs in glioblastoma patient surgery material using a clinically concordant and single-cell resolved platform. Profiling more than 2,500 ex vivo drug responses across 27 patients and 132 drugs identified class-diverse neuroactive drugs with potent anti-glioblastoma efficacy that were validated across model systems. Interpretable molecular machine learning of drug–target networks revealed neuroactive convergence on AP-1/BTG-driven glioblastoma suppression, enabling expanded in silico screening of more than 1 million compounds with high patient validation accuracy. Deep multimodal profiling confirmed Ca^2+^-driven AP-1/BTG-pathway induction as a neuro-oncological glioblastoma vulnerability, epitomized by the anti-depressant vortioxetine synergizing with current standard-of-care chemotherapies in vivo. These findings establish an actionable framework for glioblastoma treatment rooted in its neural etiology.

## Abstract (web summary)

A single-cell ex vivo screening of repurposable drugs in glioblastoma and machine learning of drug–target networks show that anti-tumor neuroactive drugs converge on the AP-1/BTG pathway, based on which prediction models and experimental in vivo and in silico validation identify the anti-depressant vortioxetine as a potential therapeutic agent.

## Main

Glioblastoma is the deadliest primary brain cancer with limited treatment options, shaped by heterogeneous developmental programs, genetic drivers and tumor microenvironments (TMEs)^1–6^. Despite an increasing understanding of this heterogeneity, the alkylating agent temozolomide (TMZ), prolonging median survival from 12 months to 15 months, remains the only first-line drug approved for glioblastoma^7,8^. Targeted therapies have been largely unsuccessful, in part due to the blood–brain barrier (BBB) limiting tumor accessibility, the presence of treatment-resistant glioblastoma stem cells (GSCs) and the lack of clinically predictive patient model systems^9–11^. Systemically addressing these therapeutic roadblocks is an urgent clinical need.

An emerging paradigm is to consider the neurobiology of glioblastoma, including stemness signatures resembling neural development^3,4,12–17^, synaptic integration of cancer cells into neural circuits^18–25^ and the modulation of specific neurotransmitter or other secretory pathways in the TME^18,26–31^. Such neural aspects of glioblastoma offer clinically actionable vulnerabilities that may be pharmacologically targeted by repurposing approved ‘neuroactive’ drugs (NADs) designed to cross the BBB and routinely prescribed for other neurological indications. Exciting recent studies have reported tumor-extrinsic modulation via the brain–glioma interface as well as unexpected roles of certain metabolic and stemness pathways in gliomas that can be targeted by specific NADs^21–25,27,30^. However, for the vast majority of NADs, their anti-cancer activity has not been tested in prospective glioblastoma patient cohorts, and tumor-intrinsic NAD targets remain incompletely mapped. Therefore, a systematic preclinical evaluation of neurotherapeutic glioblastoma vulnerabilities and personalized treatment opportunities is needed.

## Results

### Clinically concordant ex vivo drug profiling for glioblastoma

**Fig. 1:**
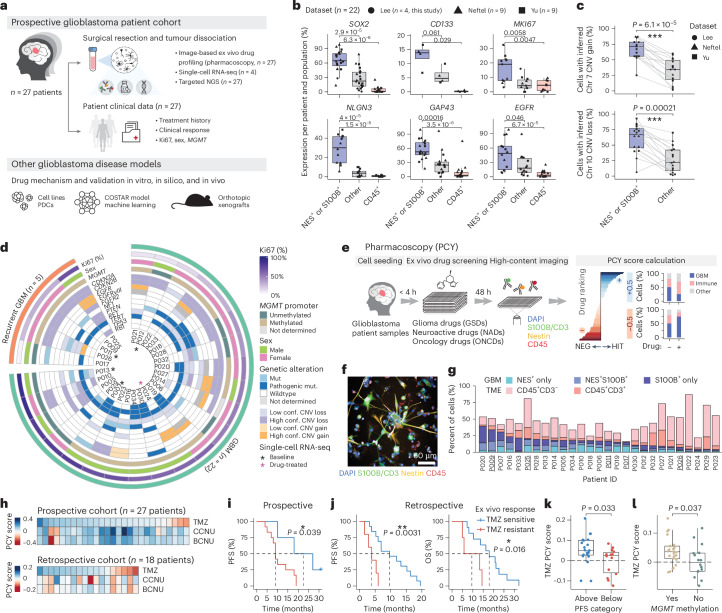
Clinical concordance of single-cell ex vivo drug profiling for glioblastoma. **a**, Prospective multimodal profiling of a glioblastoma patient cohort (*n* = 27 patients) and diverse glioblastoma disease models. Patient numbers are indicated per data type. **b**, Percent of cells expressing each gene (*y* axis) per subpopulation (*x a*xis; *n* = 22 patients; data points; shape indicates scRNA-seq dataset). *P* values were calculated by two-tailed Wilcoxon test. Box plots show 25th–75th percentiles with a line at the median; whiskers extend to 1.5 times the interquartile range. **c**, Inferred CNA analysis based on scRNA-seq datasets in **b**. Matched patient samples are connected by gray lines. Patients with less than 5% of cells with detected CNAs are excluded. **d**, Overview of the prospective cohort (*n* = 27 patients). See Supplementary Table 1 for full cohort information. conf., confidence. **e**, Real-time image-based ex vivo drug screening (PCY) workflow of glioblastoma patient samples. **f**, Example IF image of a glioblastoma patient sample (P040; scale bar, 60 µm). **g**, Baseline cellular composition across the prospective glioblastoma cohort measured by PCY. Underlines indicate patients with recurrent glioblastoma. **h**, GSD (rows; *n* = 3 drugs) response across patient samples (columns). GSD response is averaged across concentrations for TMZ and lomustine/carmustine (CCNU and BCNU, respectively). **i**,**j**, Stratification of newly diagnosed glioblastoma patient survival based on ex vivo TMZ sensitivity of (Nestin^+^/S100B^+^ and CD45^−^) cells (blue, TMZ sensitive; red, TMZ resistant). Kaplan–Meier survival curves are compared using the log-rank (Mantel–Cox) test, and the optimal TMZ PCY score cutpoint to stratify patients was determined by maximally selected rank statistics. **i**, PFS of the prospective glioblastoma cohort (*n* = 16 annotated patients) stratified by TMZ PCY score (100 µM). Tick mark indicates ongoing response. **j**, PFS (left) and OS (right) of the retrospective cohort (*n* = 18 patients) stratified by mean TMZ PCY score. **k**, TMZ PCY scores (dots; *n* = 34 patients across both cohorts) stratified by clinically reported median PFS^7^ to first-line TMZ chemoradiotherapy. Wilcoxon test. **l**, TMZ (50 µM) PCY scores across both cohorts (dots; *n* = 41 patients) stratified by *MGMT* promoter methylation status. Wilcoxon test. Box plots as in **b**. GBM, glioblastoma. Source data

To identify clinically actionable therapeutic vulnerabilities of glioblastoma, we performed prospective multimodal drug profiling across IDH-wildtype glioblastoma patient samples, two-dimensional (2D) and three-dimensional (3D) patient-derived cell (PDC) cultures, machine learning–based drug–target networks and orthotopic mouse models (Fig. 1a and Supplementary Tables 1 and 2). We adapted pharmacoscopy (PCY), an ex vivo image-based drug screening platform previously validated in functional precision medicine trials for hematological malignancies^32–35^, for the functional characterization of patient glioblastoma tissues. For both solid tumors and blood cancers, PCY identifies ‘on-target’ drug responses by quantifying the drug-induced specific reduction of cancer cells relative to non-malignant TME cells based on immunofluorescence (IF) staining. We, therefore, first set out to define and validate a clinically relevant marker profile that would capture the majority of glioblastoma cells across patient samples.

Glioblastoma cells adopt a spectrum of malignant cellular programs recapitulating neural differentiation, ranging from neural progenitor-like GSCs to more mature astrocyte-like cellular states^3,4,9,14,15,36^. As a consequence, neural progenitor markers (for example, Nestin) and astrocyte lineage markers (for example, S100B and GFAP) are widely used to characterize patient tumors^19,21,22,24,37–39^, with Nestin^+^ GSCs representing a treatment-resistant subpopulation that sustains long-term tumor growth^9–11,16^.

**Extended Data Fig. 1:**
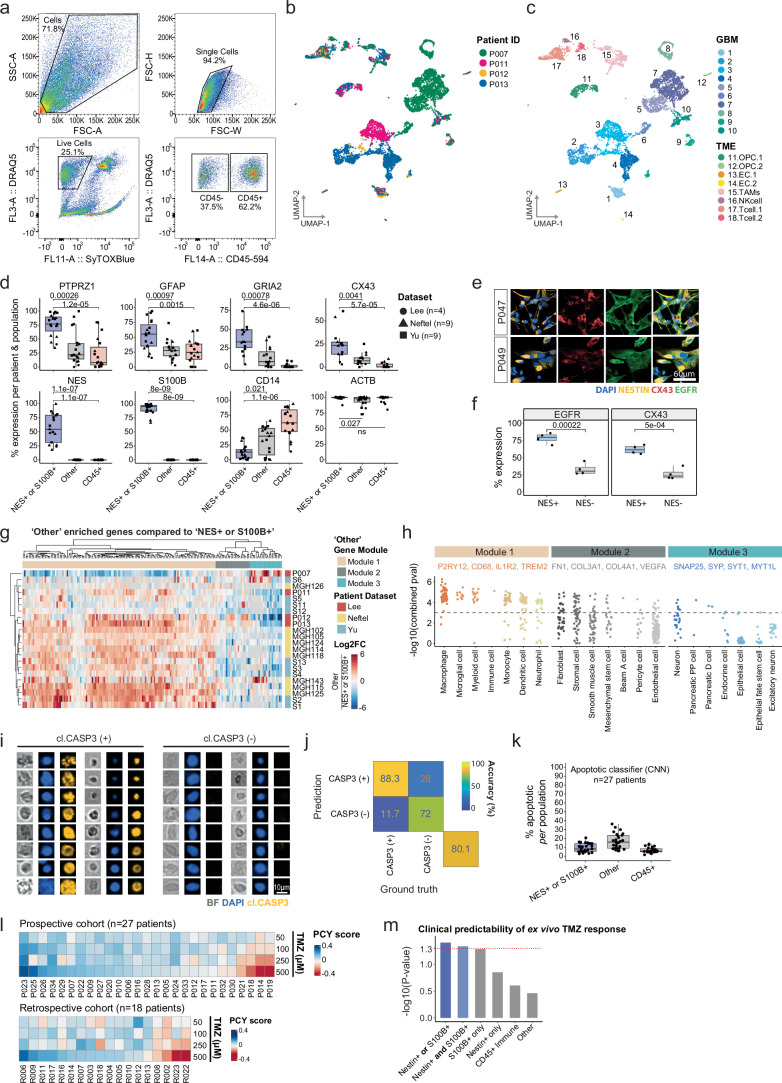
Single-cell RNA-Seq analysis and ex vivo drug profiling of standard-of-care treatment for glioblastoma. **a**, Example FACS gates of patient sample P011 to enrich for glioblastoma cells prior to scRNA-Seq (n = 50,000 cells shown). **b, c**, UMAP projection of 7684 single-cell transcriptomes colored by **b**, patient (P007: 3,475 cells; P011: 1,490 cells; P012: 330 cells; P013: 2,389 cells, *this study*), and **c**, cluster-id. TME, tumor microenvironment; OPC, oligodendrocyte precursor cells; EC, endothelial cell; TAM, tumor-associated macrophage; NK, natural killer cell. **d**, % cells expressing genes (y-axis) per patient (data points) and subpopulation (x-axis) across 22 glioblastoma patient samples (dots) and 3 scRNA-Seq datasets (shape). **e**, Example IF images of patient samples (P047, P049) labeled with different glioblastoma markers (Nestin, EGFR, and CX43). **f**, Quantification of IF images in ***e*** across n = 4 glioblastoma patient samples (dots) for EGFR and CX43 expression in either Nestin+ or Nestin- cells. Two-tailed t-test. **g**, Genes (columns) enriched in (*NES*-, *S100B*-, and *CD45*-) triple-negative cells (‘Other’) compared to ([*NES*+ or *S100B* + ] and *CD45*-) cells across 22 patients (rows) from three scRNA-seq cohorts. Heatmap depicts log2(fold change) of genes enriched in ‘Other’ cells. Expression of top-10 genes (columns) per patient (rows) clustered into 3 gene modules. **h**, Cell-type specific enrichment analysis (Web-CSEA^69^) of the ‘Other’ enriched gene modules as in ***g***. Dots represent individual Web-CSEA datasets, example member genes of their respective gene modules annotated above. **i**, Example single-cell crops of cleaved CASP3 + /- negative cells by IF in the image dataset used to train a convolutional neural network (CNN) based on nuclear (DAPI) and cell morphology (Brightfield) to detect apoptotic cells. **j**, Apoptotic classifier CNN performance in classifying the test image dataset (n = 1,214 single-cell crops). **k**, % cells classified as apoptotic by the CNN across the prospective cohort (n = 27 patients) and marker defined populations. **l**, Temozolomide PCY score (TMZ; rows; n = 4 concentrations) across patient samples (columns; prospective cohort, n = 27; retrospective cohort, n = 18). Color indicates the PCY score for glioblastoma cells. Values beyond color scale limits set to minimum and maximum values. **m**, Clinical predictability of *ex vivo* TMZ response (averaged across n = 4 concentrations) in stratifying progression free survival (PFS) of the prospective cohort (n = 16 patients). *P*-values from survival curve comparison by the log-rank (Mantel-Cox) test. **d,f,k**, Boxplots as in Fig. 1b. Source data

Analysis of 25,510 single-cell transcriptomes spanning three independent single-cell RNA sequencing (scRNA-seq) datasets and 22 patients (including four from this study) confirmed that glioblastoma cells defined by Nestin/S100B expression and absence of immune marker CD45 capture the majority of malignant cells (Fig. 1b,c and Extended Data Fig. 1a–d). These cells displayed the highest expression of markers associated with malignancy (for example, *SOX2*, *CD133*, *EGFR* and *Ki67*) in comparison to CD45^+^ immune cells and cells triple-negative for Nestin, S100B and CD45 (referred to as ‘other’ cells; Fig. 1b and Extended Data Fig. 1d). Glioblastoma cells also expressed the highest level of markers attributed to the neural properties of glioblastoma, such as synaptic circuit integration, neuronal activity-regulated paracrine signaling and tumor microtube formation (Fig. 1b and Extended Data Fig. 1d). Additional IF staining of patient samples confirmed that Nestin^+^ cells had higher expression of these malignancy and neural properties-associated markers (Extended Data Fig. 1e,f). Furthermore, inferred chromosomal copy number alteration (CNA) analysis of hallmark genetic alterations in glioblastoma confirmed the Nestin/S100B and CD45^−^ marker definition to capture the majority of malignant cells of patients in which these hallmark CNAs were detected (Fig. 1c). Lastly, cell-type-specific enrichment analysis of the triple-negative ‘other’ cells revealed additional TME cell types, including CD45-low tumor-associated macrophages/microglia, fibroblasts and stromal cells (Extended Data Fig. 1g,h).

To evaluate the clinical concordance of glioblastoma drug response profiling defined by this marker panel, we measured ex vivo responses to first-line and second-line glioblastoma chemotherapies by PCY in prospectively sampled surgery material from 27 patients with IDH-wildtype glioblastoma (‘prospective cohort’; *n* = 27; Fig. 1d and Supplementary Tables 1 and 2). Each patient sample was dissociated on the day of surgery and directly incubated with drugs for 48 h (Fig. 1e). Subsequent IF staining of the marker panel and imaging by automated microscopy revealed a high degree of inter-tumor and intra-tumor heterogeneity at baseline (Fig. 1f,g): across patients, glioblastoma cells ranged from 4% to 39%, immune cells from 1% to 82% and ‘other’ cells from 13% to 84% (Fig. 1g). In the absence of drug treatment, on average, less than 10% of glioblastoma cells were apoptotic at 48 h (Extended Data Fig. 1i–k).

We next quantified the drug-induced ‘on-target’ tumor reduction, where a positive PCY score indicates a greater reduction of glioblastoma cells relative to TME cells. Limiting our analysis to newly diagnosed patients who received TMZ as part of their first-line treatment in the clinic and with documented clinical outcome (16 of 27 patients), we found that higher ex vivo TMZ sensitivity of glioblastoma cells, but not of immune or other cells, was associated with improved patient outcome (Fig. 1h,i and Extended Data Fig. 1l,m). This clinical association was validated in a retrospective cohort (*n* = 18 biobanked samples), where higher ex vivo TMZ sensitivity of glioblastoma cells was prognostic for longer progression-free survival (PFS) and overall survival (OS) (Fig. 1j). Inversely, across both cohorts, stratification by previously reported median PFS for TMZ chemoradiotherapy (6.9 months^7^) revealed higher ex vivo TMZ sensitivities in patients with longer survival (Fig. 1k). Lastly, methylated *MGMT* promoter status was associated with higher ex vivo TMZ sensitivities, recapitulating this well-established prognostic factor (Fig. 1l). Taken together, these results demonstrate the utility of PCY for therapeutic discovery and patient stratification in glioblastoma.

### Select NADs display robust anti-glioblastoma activity

**Fig. 2:**
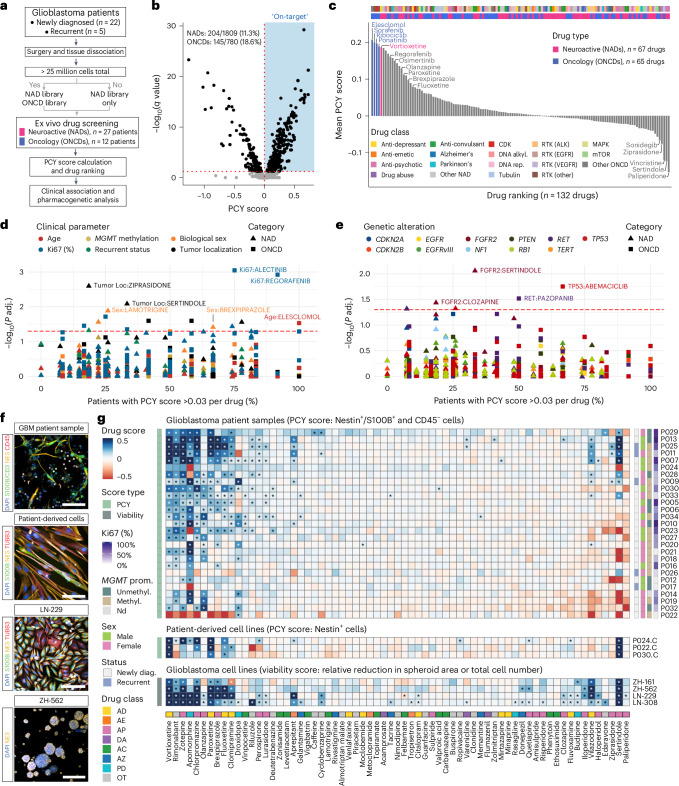
PCY identifies repurposable NADs with tumor-intrinsic anti-glioblastoma activity. **a**, PCY overview for screening neuroactive (NAD) and oncological (ONCD) drug libraries across the prospective patient cohort (*n* = 27 patients) ex vivo. **b**, Volcano plot of all measured glioblastoma PCY scores and corresponding significance (FDR-adjusted *q* value, Student’s two-tailed *t*-test). ‘On-target’ responses (blue; PCY score > 0, −log10(*q* value) > 1.3) per drug library are indicated. **c**, Drug ranking (*n* = 132 drugs) by mean PCY scores across patients. alkyl., alkylation; rep., replication. **d**, Relationship between clinical parameters and PCY score across NADs and ONCDs. Each datapoint represents a [clinical parameter:drug] association. **e**, As in **d** but for genetic alterations. **d**,**e**, Colored by clinical parameter/gene, and shape denotes drug category. Red dashed line, significance threshold. Adjusted *P* values were calculated by Wilcoxon test for two groups and by Kruskal–Wallis test for three or more, excluding cases where any category was present in fewer than three patients. **f**, Example patient sample image (P040; scale bar, 100 µm), PDC line (P040.PDC; scale bar, 100 µm), adherent glioblastoma cell line (LN-229; scale bar, 150 µm) and glioblastoma-initiating cell line (ZH-562; scale bar, 250 µm). Stains are indicated in their respective colors. **g**, NAD score matrix (*n* = 67 drugs; columns) across patient samples (*n* = 27; rows), PDC lines (*n* = 3; patient ID followed by ‘.C’) and glioblastoma cell lines (*n* = 4). Drug score (color scale) indicates the PCY score for patient samples and PDC lines (one-tailed *t*-test) or viability score for glioblastoma cell lines (two-tailed *t*-test). Values beyond color scale limits were set to either minimum or maximum values. For clinical and drug annotations, see Supplementary Tables 1 and 2. *FDR-adjusted *P* < 0.05. Source data

**Extended Data Fig. 2:**
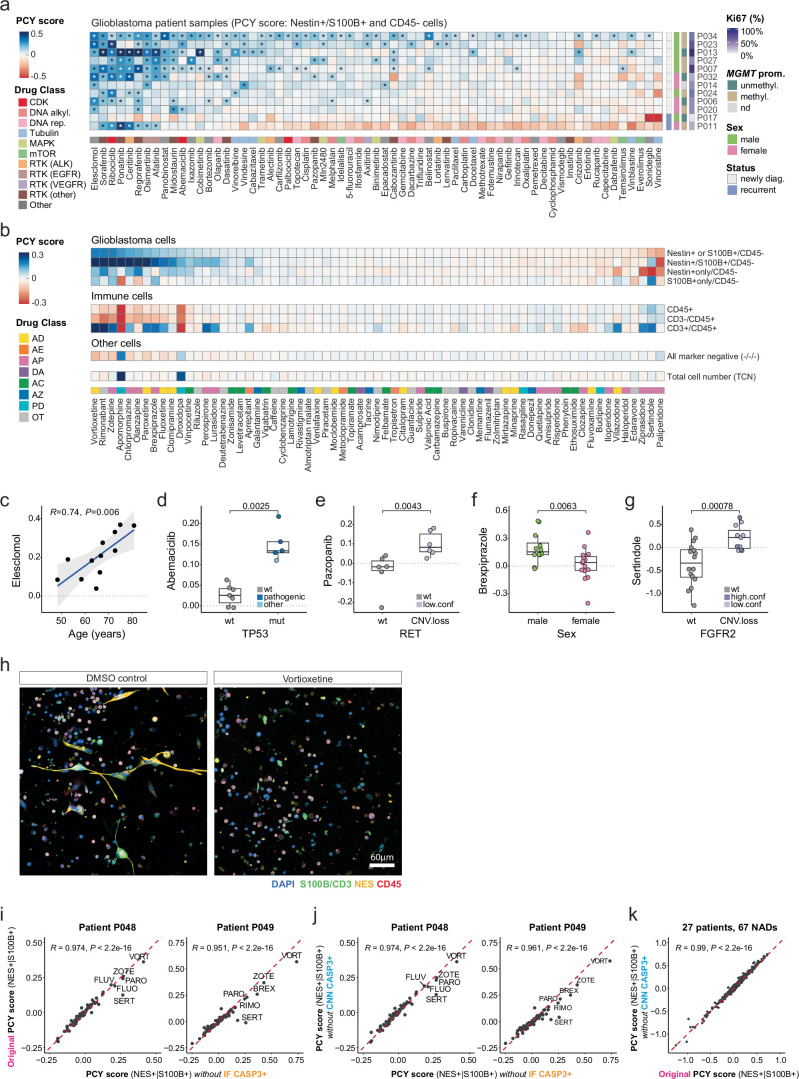
Real-time neuroactive and oncology drug screening in samples from patients with glioblastoma. **a**, PCY score matrix of oncology drugs (ONCDs; columns; n = 65 drugs) across glioblastoma patient samples (rows; n = 12 patients). Heatmap color scale indicates the PCY score of glioblastoma cells (Nestin + /S100B+ and CD45-). Asterisks (*) denote FDR-adjusted *P* < 0.05 from a one-tailed t-test. **b**, PCY score matrix of neuroactive drugs (NADs, n = 67 drugs) averaged across glioblastoma patient samples (n = 27 patients) for each cell population defined by IF markers and total cell number (TCN). Heatmap color scale indicates the mean PCY score of each respective population averaged across patients. **a, b**, Outliers beyond color scale limits were correspondingly set to minimum and maximum values. For clinical and drug annotations, see Supplementary Tables 1 and 2. **c-g** Glioblastoma PCY scores (y-axis) plotted per patient against selected parameters (x-axis). **c**, Age versus Elesclomol response. Linear regression line with a 95% confidence interval. Pearson correlation coefficient with two-tailed *P*-value annotated. **d**, *TP53* mutational status versus Abemaciclib response. **e**, *RET* mutational status versus Pazopanib response. **f**, Biological sex versus Brexpiprazole response. **g**, *FGFR2* copy number loss versus Sertindole response. Conf: confidence. **h**, Example IF images of a patient sample (P025) at baseline (DMSO control) and treated with Vortioxetine. Scale bar, 60 µm. **i-k**, Comparison of neuroactive drug PCY scores of glioblastoma cells (n = 67 NADs; original PCY score) to NAD PCY scores calculated by excluding cleaved CASP3+ apoptotic cells. Apoptotic cells are defined either by IF (PCY score without IF CASP3 + ) or by the apoptotic CNN classifier (PCY score without CNN CASP3 + ; see also *Methods*). Pearson correlation coefficients with *P*-values annotated. **i, j**, NAD screens performed in two validation patient samples (P048, P049). **i**, Comparison of the original PCY score to the PCY score without IF CASP3+ **j**, Comparison of the PCY score without IF CASP3+ the PCY score without CNN CASP3+ **k**, Comparison of the original PCY score to the PCY score without CNN CASP3+ across the prospective cohort (n = 27 patients) and neuroactive drugs (n = 67 drugs). **d-g**, Two-tailed Wilcoxon test. Boxplots as in Fig. 1b. Source data

To find repurposable drug candidates for glioblastoma treatment, we tested both neuroactive and oncology drug libraries across patient samples by PCY (Fig. 2a–g, Extended Data Fig. 2a–g and Supplementary Table 2). The NAD library, screened across the prospective cohort (*n* = 27), consisted of drugs approved for neurological diseases such as depression, schizophrenia and Alzheimer’s disease (*n* = 67 drugs; 20 µM). In contrast, the oncology drug (ONCD) library, screened when enough surgical material was available (*n* = 12), included cancer therapies such as cyclin-dependent kinase (CDK) and receptor tyrosine kinase (RTK) inhibitors (*n* = 65 drugs; 10 µM). As before, we measured the ‘on-target’ reduction of glioblastoma cells after 48 h of drug incubation after surgery while also quantifying the drug responses of immune and ‘other’ cells (Extended Data Fig. 2b).

Across the cohort, we identified 13.5% of on-target ex vivo drug responses (349 out of 2,589 measured; PCY score > 0 and false discovery rate (FDR)-adjusted *q* < 0.05; Fig. 2b). The top four drugs were oncology drugs targeting different aspects of glioma etiology: the oxidative stress inducer elesclomol (rank 1 out of 132 drugs), tyrosine kinase inhibitors sorafenib (rank 2) and ponatinib (rank 4) and the CDK inhibitor ribociclib (rank 3). Several top ONCD candidates had reported BBB permeability, including elesclomol, EGFR inhibitor osimertinib (rank 11) and tyrosine kinase inhibitor regorafenib (rank 9). Exploring the clinical and pharmacogenetic associations with ONCD responses across patients revealed higher ex vivo sensitivity to elesclomol with age, higher sensitivity to CDK4/6 inhibitor abemaciclib in patients with *TP53* mutations and higher sensitivity to RTK inhibitor pazopanib in patients with RET copy number loss (Extended Data Fig. 2c–e). This exploratory analysis can, thus, efficiently generate hypotheses for personalized glioblastoma treatment opportunities, warranting further evaluation in larger cohorts.

NADs resulted in a similar fraction of significant on-target responses across the cohort (11.3%; Fig. 2b), with 15 NADs displaying potent anti-glioblastoma activity across patients (referred to as ‘top NADs’ or ‘PCY-hit NADs’; mean PCY score > 0.03; Fig. 2b,g and Supplementary Table 2). The top-ranking NAD was the anti-depressant vortioxetine (rank 5 overall; Fig. 2c,g and Extended Data Fig. 2h), which showed significant ex vivo efficacy in 18 out of 27 patients (66.7%). Other clinically attractive NADs included paroxetine (rank 15, 44.4% of patients) and fluoxetine (rank 19, 40.7% of patients), both anti-depressants of the selective serotonin reuptake inhibitor (SSRI) class, as well as the anti-psychotic brexpiprazole (rank 17, 48.1% of patients) (Fig. 2c,g). However, not all identified top NADs were clinically attractive, considering the reported side effects of cannabinoid receptor blocker rimonabant (rank 6) and anti-psychotic zotepine (rank 7), yet they may provide mechanistic insights. These PCY-based NAD responses were reproduced using different ways to detect apoptotic cells (Extended Data Fig. 2i–k and Methods) and were robust to tumor content, assay timepoint and culture condition (Supplementary Fig. 1). Exploring their clinical and pharmacogenetic associations revealed higher ex vivo sensitivity to brexpiprazole in males (Fig. 2d and Extended Data Fig. 2f) and higher sertindole sensitivity in patients with *FGFR2* copy number loss (Fig. 2e and Extended Data Fig. 2g).

**Extended Data Fig. 3:**
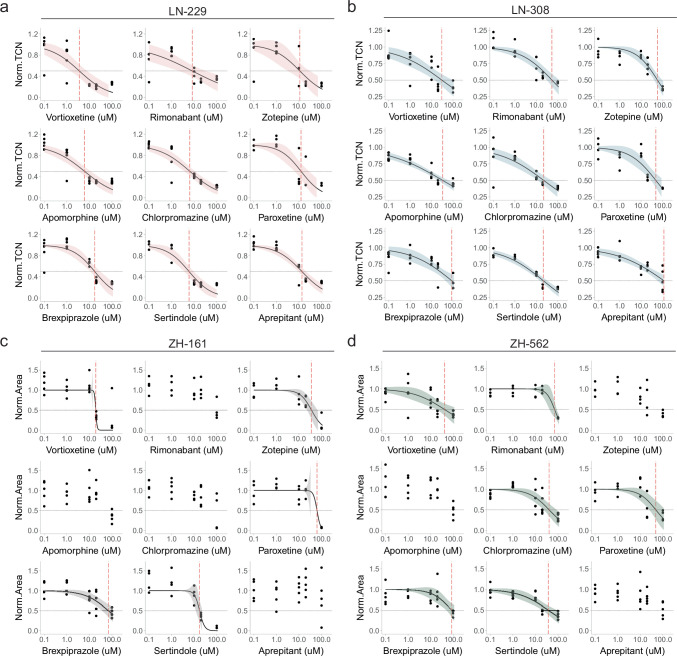
Concentration-response curves of glioblastoma cell lines. **a-d**, Concentration-response curves of glioblastoma cell lines (**a**, LN-229; **b**, LN-308; **c**, ZH-161; **d**, ZH-562) for a subset of neuroactive drugs (n = 9 drugs) across different concentrations (logarithmically spaced x-axis, n = 5 concentrations). **a, b**, Y-axis denotes relative cell counts or **c, d**, relative 2D-projected spheroid area for 3D cultures normalized to DMSO control. Concentration-response curves (solid black lines) are fitted when possible with a two-parameter log-logistic distribution with 95% confidence intervals (shaded per cell line) and ED50 (red dashed lines). n = 3-5 replicate wells/drug (dots), n = 15 DMSO wells. Source data

We tested the NAD library in additional glioblastoma disease models, including PDCs (*n* = 3 lines; Fig. 2f,g) and commonly used 2D and 3D glioblastoma cell lines (*n* = 4 lines; Fig. 2f,g). Top NADs effectively reduced fractions of Nestin^+^ cells and metabolic activity in PDCs, total cell numbers in adherent cell lines (LN-229 and LN-308) and spheroid size in glioblastoma-initiating cell lines (ZH-161 and ZH-562), with confirmed concentration–response relationships (Fig. 2g, Extended Data Fig. 3a–d and Supplementary Fig. 2). The efficacy of anti-depressants vortioxetine, paroxetine and fluoxetine were exceptionally consistent, where vortioxetine was the top-ranking NAD across all model systems tested (Fig. 2g). Thus, by comprehensively screening across glioblastoma patient surgery material and model systems, we identified a set of repurposable NADs with potent anti-glioblastoma efficacy. The consistency of these top NADs across model systems, even in the absence of the TME and synaptic circuitry, indicates the presence of one or more tumor-intrinsic neural vulnerabilities.

### Divergent functional dependencies on NAD targets

**Fig. 3:**
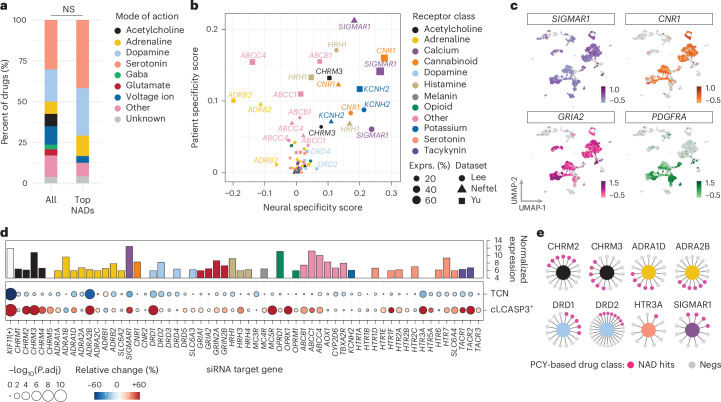
Divergent genetic dependencies on canonical primary target genes of NADs. **a**, Drug mode of action for all NADs (*n* = 67 drugs; left) and top NAD hits (*n* = 15 drugs with a mean patient PCY score > 0.03; right) represented as stacked bar plots. NS, not significant by hypergeometric enrichment test. **b**, NAD PTG expression in 22 glioblastoma patient samples across three scRNA-seq datasets (shape) plotted as the neural specificity score (*x* axis) versus patient specificity score (*y* axis) for each PTG (dot, gene; size, percent expression; color, receptor class). **c**, scRNA-seq log_10_(expression) of selected neuroactive PTGs (*SIGMAR1*, *CNR1* and *GRIA2*) and oncogenic RTK (*PDGFRA*) visualized on the UMAP projection, as in Extended Data Fig. 1b. **d**, Baseline RNA-seq expression (top panel; *y* axis; color, receptor class) as in **b** and siRNA-mediated gene silencing of PTGs in LN-229 cells (*n* = 59 siRNA conditions; columns; bottom panel). Total cell number (TCN) reduction and cleaved CASP3^+^ fraction increase (cl.CASP3^+^) relative to the (−) control *FLUC* siRNA condition depicted as a circle per gene. Two-tailed *t*-test where circle sizes scale with the −log_10_(FDR-adjusted *P* value), and color represents relative change for each tested PTG. **e**, Example PTGs with genetic dependencies (core nodes) linking to both PCY-hit (pink; NAD hits) and PCY-negative (gray; Negs) drugs. PTGs are colored according to receptor class as in **b**. Source data

**Extended Data Fig. 4:**
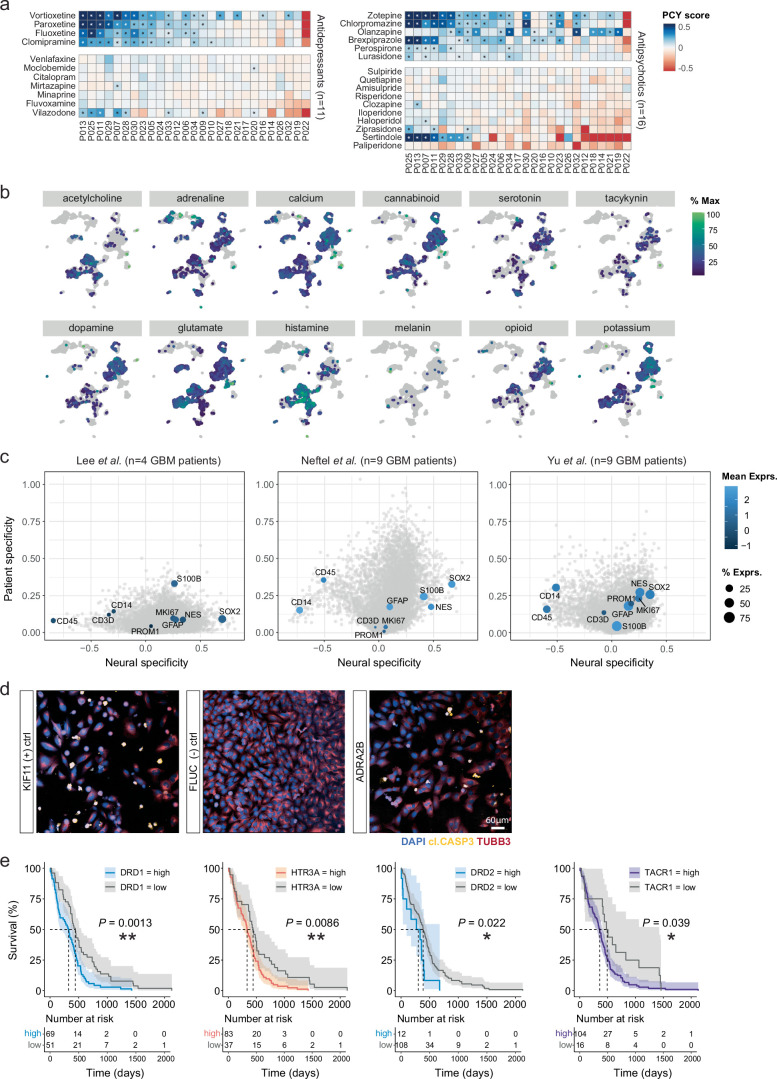
Functional genetic dependencies of glioblastoma on heterogeneously expressed neuroactive drug targets. **a**, PCY score matrix of antidepressants (left, n = 11 drugs) and antipsychotics (right, n = 16 drugs) across glioblastoma patient samples (n = 27 patients) subsetted from the original matrix, as shown in Fig. 2g. **b**, UMAP projection of 7684 single-cell transcriptomes from four glioblastoma patient samples (P007, P011, P012, P013), colored by aggregate scRNA-Seq expression across primary target genes (PTG) per receptor class in Fig. 3b. Color scaled to percent of maximum expression per receptor class. **c**, Neural specificity score (x-axis) versus patient specificity score (y-axis) for three independent glioblastoma scRNA-Seq datasets. Each dot represents a gene, with key marker genes annotated with labels. Key marker genes colored by mean detected expression across cells and dot size scales with percent of expressed cells. All other detected genes are colored in grey. (Lee et al., *this study*; n = 4 patients, n = 7684 cells, n = 15,668 genes; Neftel et al., n = 9 patients, n = 13,519 cells, n = 22,160 genes; Yu et al., n = 9 patients, n = 4307 cells, n = 19,098 genes). **d**, Example IF images of siRNA-mediated gene silencing of the positive control gene (*KIF11* (+) ctrl; left), negative control gene (*FLUC* (-) ctrl; middle), and *ADRA2B* (right). Scale bar, 60 µm. Cells are stained for DAPI (blue), cleaved CASP3 (yellow) and TUBB3 (red). **e**, Kaplan-Meier survival analysis and associated risk tables of the TCGA primary glioblastoma cohort (n = 120 patients) based on RNA-Seq expression of 4 PTGs (panels) that significantly reduce cell viability in Fig. 3d and stratify patient survival. Optimal cut-point for patient stratification (high, low) is determined by maximally selected rank statistics. Survival curves are compared using the log-rank (Mantel-Cox) test. 95% confidence intervals of Kaplan-Meier estimates are indicated in shaded curves. Source data

The NADs with anti-glioblastoma efficacy represented diverse drug classes, indicating that canonical mode of action did not explain their efficacy (Fig. 3a). Among our tested serotonin and dopamine pathway modulators, for example, only four out of 11 anti-depressants (36%) and six out of 16 anti-psychotics (38%) exhibited anti-glioblastoma activity in patient samples (Extended Data Fig. 4a). Such drug classifications, however, simplify the polypharmacological drug–target profiles of NADs. Most NADs act on multiple primary target genes (PTGs), including ion channels and G-protein-coupled receptors (GPCRs), whose expression remains a largely unexplored dimension of glioblastoma heterogeneity.

To this end, we evaluated NAD PTG expression profiles across the three glioblastoma scRNA-seq datasets (Fig. 3b,c and Extended Data Fig. 4b,c)^4,40^. Among PTGs with reported biochemical interactions with NADs (based on the Drug Targets Commons (DTC)^41^), expression of potassium channels, glutamate receptors and cannabinoid receptors were enriched in glioblastoma cells, whereas other target classes showed broader expression patterns (Extended Data Fig. 4b). To characterize PTG expression heterogeneity, we calculated neural specificity and patient specificity scores (Fig. 3b, Extended Data Fig. 4c, Supplementary Table 3 and Methods), where a higher neural specificity indicates relative enrichment in neural lineage cells (range −1 to 1), and a higher patient specificity (range 0 to 1) indicates more patient-specific expression. Gene transcripts encoding ion channels and receptors with high neural specificity included the calcium signaling modulator *SIGMAR1* and cannabinoid receptor *CNR1*. Both had considerably lower patient specificity than oncogenic RTKs *EGFR* and *PDGFRA*, despite similar detection levels (Fig. 3c and Supplementary Table 3), highlighting consistent pan-patient expression of NAD targets in glioblastoma.

We tested the dependency on these NAD PTGs by performing a reverse genetic screen in LN-229 glioblastoma cells (*n* = 59 genes; Fig. 3d, Extended Data Fig. 4d and Supplementary Table 4) with similar PTG expression and NAD sensitivities to patient samples (Figs. 2g and 3d). Knockdown of nine PTGs significantly decreased cell viability (Fig. 3d and Extended Data Fig. 4d), of which lower expression levels of *DRD1*, *DRD2*, *HTR3A* and *TACR1* were also associated with better patient survival in The Cancer Genome Atlas (TCGA) glioblastoma cohort (Extended Data Fig. 4e). However, these PTG dependencies were predominantly targeted by NADs without anti-glioblastoma activity by PCY. For example, only five of the 16 *DRD1*-targeting NADs, and only one out of 11 *HTR3A*-targeting NADs, were PCY-hits (Fig. 3e). Therefore, although presenting possible neural vulnerabilities, these genetic PTG dependencies are unlikely to explain the anti-glioblastoma activity of our top NADs.

### Drug–target network convergence predicts NAD efficacy

Despite their chemical and primary target diversity, top NADs may converge upon common downstream signaling pathways. To test this, we developed an interpretable machine learning approach that searches for ‘convergence of secondary drug targets analyzed by regularized regression’ (COSTAR). COSTAR is designed to identify the minimal drug–target connectivity signature that is maximally predictive of patient drug efficacy (Methods).

**Fig. 4:**
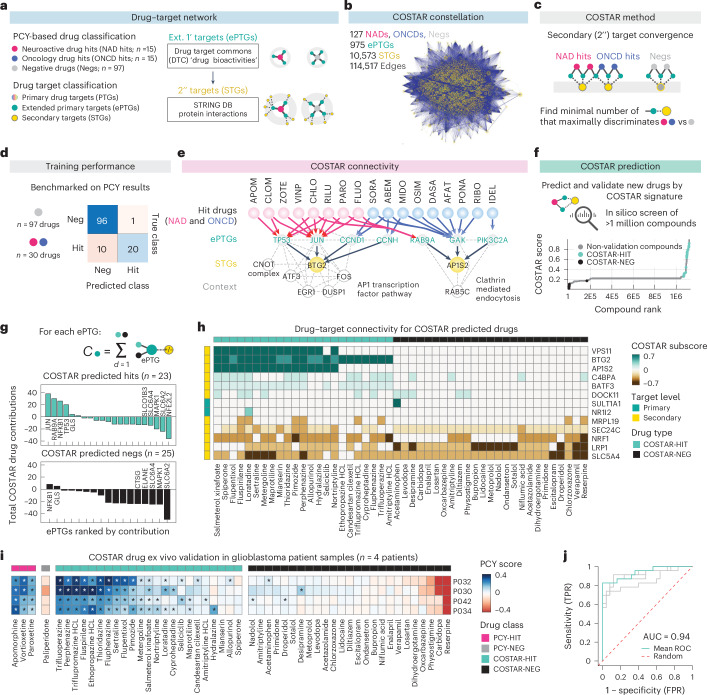
Molecular convergence on a neuroactive drug–target connectivity signature predictive of anti-glioblastoma efficacy. **a**, COSTAR workflow. **b**, COSTAR network of 127 PCY-tested drugs, 965 ePTGs and 10,573 STGs, connected by 114,517 edges. **c**, COSTAR method by logistic LASSO regression. See also Methods. **d**, COSTAR training model performance compared to PCY-based experimental ground truth. **e**, COSTAR connectivity (solid lines) reveals convergence of NAD (pink) and ONCD (blue) hits to key ePTGs (gray) and STGs (yellow). See Extended Data Fig. 5c for the full model. Additional proteins (white nodes) with high-confidence interactions to STGs (dashed lines) are shown. **f**, In silico drug screen across 1,120,823 compounds by COSTAR. Compounds are ranked (*x* axis) by their predicted PCY-hit probability (COSTAR score; *y* axis). Predicted drug hits (COSTAR-HIT; mint green) and predicted non-hits (COSTAR-NEG; black) selected for experimental validation are indicated. **g**, ePTGs (*x* axis) ranked by their integrated contribution ‘*C*’ to predict a hit (+1) or a non-hit (−1) (*y* axis) in the COSTAR model, separated for COSTAR-HITs (top) and COSTAR-NEGs (bottom) (‘*d*’). **h**, Drug–target connectivity of select COSTAR-predicted drugs (columns; *n* = 23 COSTAR-HIT drugs; *n* = 25 COSTAR-NEG drugs) to primary and secondary drug targets (rows). COSTAR subscore (heatmap color scale) is the LASSO model coefficient multiplied by the integrated connectivity of drug to target mapping. Target genes with absolute COSTAR LASSO coefficients greater than 0.1 are displayed. **i**, Experimental ex vivo validation by PCY of COSTAR-HIT (*n* = 23; mint green) and COSTAR-NEG (*n* = 25; black) drugs (columns) across four glioblastoma patient samples (rows) including positive (PCY-hits; pink; *n* = 3) and negative (PCY-negative; dark gray; *n* = 1) control drugs. Heatmap color scale indicates the PCY score of glioblastoma cells. One-tailed *t*-test; *FDR-adjusted *P* < 0.05. Outliers beyond color scale limits are set to minimum and maximum values. **j**, Receiver operating characteristic (ROC) curves (gray, *n* = 4 patients; mint green, mean across patients; red dashed, random classifier) describing the COSTAR validation accuracy in glioblastoma patient samples of the COSTAR-predicted drugs (*n* = 48 drugs; corresponding to **i**). FPR, false-positive rate; PCY-HIT, PCY-hit; PCY-NEG, PCY-negative; TPR, true-positive rate. Source data

**Extended Data Fig. 5:**
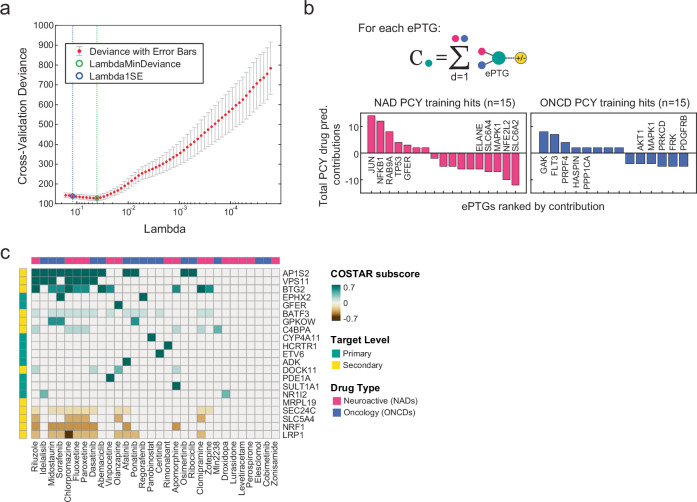
Drug-target connectivity identified by COSTAR. **a**, Visualization of the local optimum in the cross-validated predictive power of COSTAR LASSO regression when fitting a binomial model to predict drug activity by PCY (hit vs neg) based on a drug’s connectivity pattern (COSTAR constellation, shown in Fig. 4b). X-axis denotes the Lambda regularization parameter (n = 60 unique values) and the y-axis denotes the cross-validated error of the model (deviance) across independent bootstrapped runs (n = 20 runs). Red dots (average) and light grey error bars (standard deviation) are indicated. Vertical dashed lines and colored circles indicate either the Lambda value with the minimal mean squared error (green, MSE) or the more conservative Lambda value with minimal MSE plus one standard deviation (blue, MSE + 1STD). **b**, ePTGs (x-axis) ranked by their integrated contribution ‘C’ to predict a hit (+1) or non-hit (-1) (y-axis) in the COSTAR model, separated for PCY-hit NADs (left) and ONCD (right). **c**, Drug-target connectivity of PCY-hit drugs that were part of the COSTAR training data (columns; n = 30 drugs) to primary and secondary drug targets (rows). COSTAR subscore (heatmap color scale) is the LASSO model coefficient multiplied by the integrated connectivity of drug-to-target mapping. Target genes with absolute COSTAR LASSO coefficients >0.1 are displayed. Target level (primary or secondary target) is annotated per gene on the left. Source data

We extended the drug–target search space to include PTGs with any bioactivity annotated by DTC (extended primary target genes (ePTGs); Fig. 4a) and their secondary target genes (STGs) based on protein–protein interactions (STRING database; Fig. 4a). This resulted in a drug–target connectivity map, or ‘COSTAR constellation’, of all DTC-annotated drugs in our NAD and ONCD libraries (*n* = 127 of 132 drugs) with 975 ePTGs, 10,573 STGs and 114,517 edges (Fig. 4b). Using logistic LASSO regression, we trained a model that identifies the minimal set of STGs that maximally discriminates PCY-hit drugs (*n* = 30; top 15 from both drug libraries) from PCY-negative drugs (*n* = 97) in a cross-validation setting (Fig. 4c and Extended Data Fig. 5a). Thereby, COSTAR converged upon the minimal connectivity signature that was predictive of ex vivo anti-glioblastoma drug efficacy (Fig. 4a–e and Extended Data Fig. 5a–c). COSTAR identified a signature that classified the 127 drugs with 92.1% accuracy, correctly predicting 20 of 30 PCY-hit drugs and 96 of 97 PCY-negative drugs (Fig. 4d).

The COSTAR connectivity signature linked PCY-hit NADs to the secondary target BTG2, predominantly through JUN and TP53 ePTGs (Fig. 4e and Extended Data Fig. 5b,c). BTG2 and TP53 are both tumor suppressors that control cell cycle and differentiation, whereas JUN is a member of the AP-1 transcription factor (TF) family that, in a neural context, regulates gene expression and apoptosis in response to stimuli, such as neural activity or insult^42^. Conversely, most PCY-hit ONCDs were connected to the secondary target AP1S2, a protein involved in clathrin coat assembly, through the cyclin G-associated kinase GAK (Fig. 4e and Extended Data Fig. 5b,c). Taken together, this reveals therapeutic pathway convergence on AP-1 TFs and cell cycle regulation as a unique signature predictive of anti-glioblastoma activity of NADs.

COSTAR can compute the hit probability of any annotated compound by matching its drug–target profile to the learned connectivity signature. To evaluate the predictive power of the COSTAR signature and find additional NAD candidates, we screened 1,120,823 DTC-annotated compounds in silico and experimentally validated 23 top-scoring and 25 bottom-scoring compounds (COSTAR-HIT and COSTAR-NEG, respectively; Fig. 4f and Supplementary Data 2). Of these, only the COSTAR-HITs were linked to the secondary target BTG2, primarily through JUN (Fig. 4g,h). We tested all 48 compounds across four glioblastoma patient samples and observed excellent agreement between COSTAR predictions and PCY scores (mean area under the curve (AUC) = 0.94; Fig. 4i,j). The confirmed COSTAR-HITs again represented diverse NAD classes, including the anti-psychotic trifluoperazine, anti-parkinsonian ethopropazine and anti-depressant sertraline (Fig. 4i). These results substantiate AP-1/BTG pathway convergence as a therapeutic signature that predicts NADs with ex vivo anti-glioblastoma activity.

### Altered tumor neurophysiology induces an anti-proliferative program

**Fig. 5:**
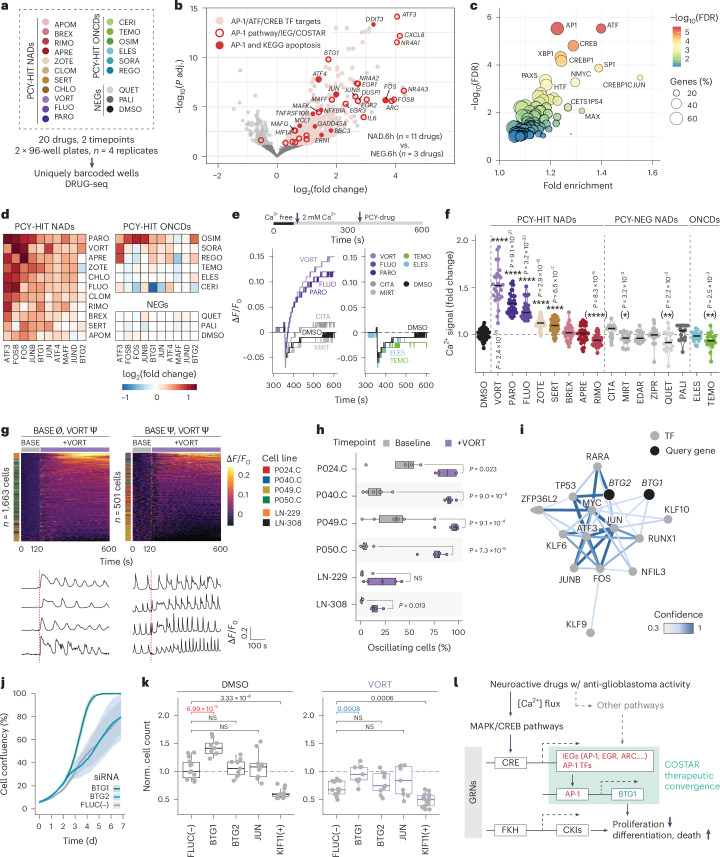
NADs alter glioblastoma neurophysiology and engage an anti-proliferative AP-1/BTG GRN. **a**, Workflow for DRUG-seq^43^ of drug-treated LN-229 cells. **b**, Transcriptional response of PCY-hit NAD-treated cells compared to NEG-treated cells (6 h; as in **a**). Significant genes by two-tailed Wald test (DESeq2) in light gray or colored according to their gene annotations (see legend). **c**, TFBS enrichment analysis of significantly upregulated genes in **b**. Circles, TF annotations. **d**, log_2_(fold change) of AP-1 TF and BTG family gene expression (columns) significantly upregulated by 6-h PCY-hit NAD (rows) treatment compared to NEG. **e**, Calcium response (Δ*F*/*F*_0_; *y* axis) over time (*x* axis) of LN-229 cells upon drug treatment. Timeline depicts FLIPR assay setup. Representative traces showing Δ*F*/*F*_0_, change in fluorescence intensity relative to baseline for NAD (left) and ONCD (right) drug conditions. **f**, Fold change in extracellular calcium influx upon drug treatment relative to DMSO measured as in **e** (*n* = 8 assay plates; *n* = 17 conditions; *n* = 18–30 wells per drug; DMSO, *n* = 47 wells). Asterisks in parentheses, median [Ca^2+^ fold change] < 0. Black line, median value. **g**, Single-cell-resolved calcium response (Δ*F*/*F*_0_) measured by ratiometric Fura-2 imaging over time at baseline (BASE) and after vortioxetine treatment (+VORT; 20 µM) across six cell lines (*n* = 3,561 cells; see also Extended Data Fig. 7c–f). Panels depict single-cell calcium responses (rows) over time (columns), stratified by the presence (Ψ) or absence (Ø) of calcium oscillations at baseline and VORT treatment. Representative single-cell traces (*n* = 4 per heatmap) are depicted below. **h**, Percent of cells displaying calcium oscillations (*x* axis) at baseline (gray) and after VORT treatment (purple) across cell lines (*y* axis; *n* = 6). Dots, independent experiments (*n* = 4–6 experiments per line). Paired two-tailed *t*-test. **i**, BTG1/2 transcriptional regulation (PathwayNet^54^). Black nodes, query genes; gray nodes, top 13 inferred TF interactions. Edge colors, relationship confidence. **j**, LN-229 confluency by live-cell imaging (*y* axis) over time (*x* axis) after gene knockdown. Mean (line) and standard deviation (bands) of *n* = 4 replicate wells are shown. **k**, LN-229 cell counts (*y* axis) after gene knockdown (columns) at baseline (left) and vortioxetine treatment (10 µM; right; *n* = 9–14 replicate wells per condition, *n* = 2 experiments). Normalized to *FLUC* at baseline. **a**,**e**,**f**, Drug abbreviations are in Supplementary Table 2. **f**,**k**, Two-tailed *t*-test. *P* values were adjusted for multiple comparisons by Holm correction. **l**, Summary diagram by which NADs target glioblastoma. CRE, cAMP response element; CKI, cyclin-dependent kinase inhibitor; FKH, forkhead binding motif. Box plots as in Fig. 1b. NS, not significant; PCY-HIT, PCY-hit; PCY-NEG, PCY-negative. Source data

**Extended Data Fig. 6:**
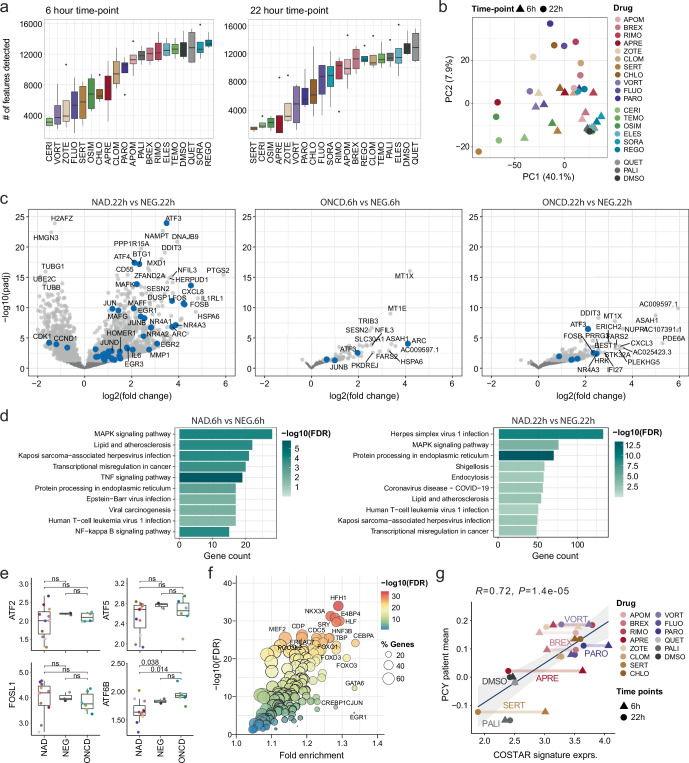
DRUG-Seq confirms an AP-1 mediated transcriptional response specific to neuroactive drugs with anti-glioblastoma efficacy. **a**, Number of features detected by DRUG-Seq (y-axis) per drug condition (columns) and by time-point n = 20 drugs, n = 2 time-points, n = 4 replicates per drug/time-point. **b**, Principal component analysis (PCA) of averaged RNA-Seq counts per drug (color) and time-point (shape). **c**, Comparisons of drug induced transcriptional profiles by DRUG-Seq shown as log2(fold change) versus –log10(adjusted *P*-value) for NADs vs NEGs (22 h, left), ONCDs vs CTRLs (6 h, middle), and ONCDs vs CTRLs (22 h, right). Significant genes by two-tailed Wald test (DESeq2) in light grey. Highlighted genes (blue) include AP-1 transcription factor (TF) network genes (PID AP1 PATHWAY^79^) and key COSTAR signature genes. **d**, Top enriched KEGG terms for differentially expressed genes based on DESeq2 comparisons of NADs vs NEGs (6 h, left) and NADs vs NEGs (22 h, right). Bars represent the number of differentially expressed genes present in the annotation, and colors indicate –log10(false discovery rate). **e**, Four AP-1 transcription factors that are down-regulated or unchanged after PCY-hit NAD treatment at 6 h. (y-axis, normalized RNA-Seq counts). Box plot groups (x-axis) correspond to drug categories and dots represent the average expression per drug (colored as in Extended Data Fig. 6b). ‘PCY-hit NAD’ and ‘PCY-hit ONCD’ abbreviated to NAD and ONCD, respectively. Two-tailed t-test. **f**, Transcription factor binding site enrichment analysis of genes that were upregulated in NAD treated cells in Extended Data Fig. 6c (22 h, left). Circles correspond to transcription factor annotations, circle sizes scale with the fraction of genes present in the annotation, and colors indicate –log10(false discovery rate). **g**, Correlation of average COSTAR signature expression (x-axis) with *ex vivo* patient neuroactive drug response (y-axis) plotted per drug (color) and time-point (shape). Mean glioblastoma PCY score across patients (n = 27 patients) of neuroactive drugs (n = 11 PCY-hit NADs, n = 3 NEGs) plotted against their corresponding geometric mean expression of AP-1 TFs and *BTG1/2* genes as shown in Fig. 5d. Linear regression line with a 95% confidence interval. Pearson correlation coefficient with two-tailed *P*-value annotated. **a**, **e**, Boxplots as in Fig. 1b. Source data

The COSTAR signature suggests a common gene regulatory network (GRN) underlying the activity of PCY-hit NADs. To confirm this, we measured the transcriptional response of LN-229 cells at 6 h and 22 h to 19 select drugs by DRUG-seq^43^ (Fig. 5a–d, Extended Data Fig. 6a–g and Supplementary Table 2). In alignment with COSTAR, differential gene expression analysis revealed a common AP-1 and BTG signature induced by diverse PCY-hit NADs (Fig. 5b,d and Extended Data Fig. 6c). This involved rapid and sustained upregulation of eight AP-1 TFs, including immediate early genes (IEGs) *JUN* and *c-FOS*, known to mediate neural activity and apoptosis^42,44–46^, and stress-induced AP-1 TFs *ATF3* and *ATF4* (Fig. 5b,d and Extended Data Fig. 6c). Conversely, downregulated AP-1 factors included *ATF5* and *ATF6B*, shown to promote glioblastoma cell survival and radioresistance, respectively^47,48^, whereas *FOSL1*, implicated in response to irradiation in glioblastoma, showed no upregulation^49^ (Extended Data Fig. 6e). Additional upregulated IEGs *NR4A1*, *EGR1* and *ARC* and MAPK pathway enrichment further implicated neural activity-like signaling (Fig. 5b and Extended Data Fig. 6d). *BTG1*, a homolog of *BTG2*, was among the top 20 most significantly upregulated genes (Fig. 5b,d and Extended Data Fig. 6c), whereas *BTG2* was particularly induced in response to vortioxetine (Fig. 5d). In contrast, tested ONCDs, including first-line chemotherapy TMZ, did not elicit this global AP-1/BTG response (Fig. 5d and Extended Data Fig. 6c). Transcription factor binding-site (TFBS) enrichment analysis of the NAD-induced genes at 6 h revealed AP-1, ATF and CREB, a calcium-activated regulator of AP-1 transcription^50^, as the most significantly enriched motifs present among 60% of upregulated genes (Fig. 5b,c and Extended Data Fig. 6f). At 22 h, expression of AP-1 factors was sustained, and forkhead TF family motifs, known to regulate long-term cell differentiation succeeding AP-1 (ref. ^51^), were enriched among the upregulated genes (Extended Data Fig. 6f).

**Extended Data Fig. 7:**
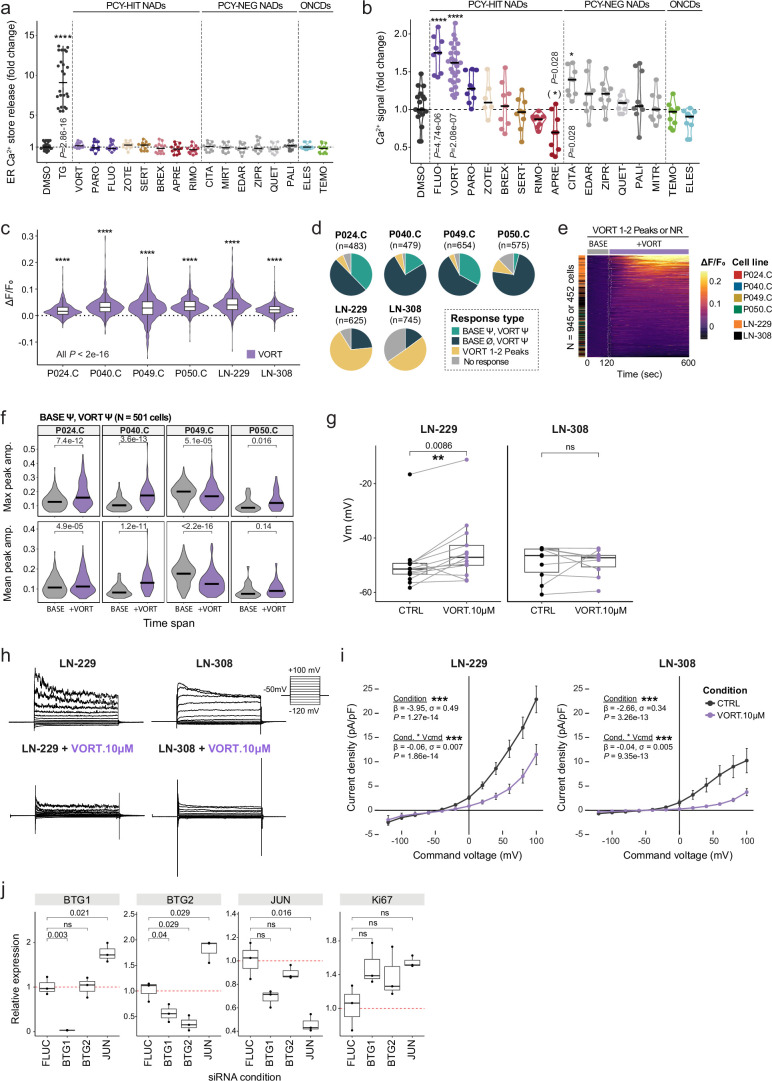
Vortioxetine induces a robust calcium response and alters the electrophysiological properties of glioblastoma cells. **a**, ER calcium store release measured by FLIPR assays in LN-229 cells (n = 4 assay plates; n = 18 conditions; n = 12 wells/drug; DMSO and Thapsigargin (TG) positive control, n = 24 wells each). **b**, Extracellular calcium influx measured by FLIPR assays in F050.C (n = 17 conditions as in Fig. 5f). (*) denote conditions where the median [Ca2+ fold change] < 0. Black line: median value. **a, b**, Fold change relative to DMSO after drug treatment. Two-tailed t-test against DMSO. *P*-values adjusted for multiple comparisons by Holm correction. Black line: median value. **c**, Single-cell-resolved calcium response (ΔF/F_0_) measured by ratiometric Fura-2 imaging across 6 cell lines (x-axis; n = 3,561 cells total). Mean change in calcium signal immediately after Vortioxtine treatment compared to baseline, each averaged across a 120 s time window. Paired (baseline vs drug treatment) two-tailed Wilcoxon test. **d**, Calcium response type stratified by the presence (Ψ) or absence (Ø) of oscillatory calcium signaling at baseline (BASE) and Vortioxetine (VORT; 20) treatment. ‘VORT 1-2 Peaks’: non-oscillatory calcium response with 1-2 peaks after VORT treatment. **e**, Heatmap of single-cell-resolved (rows) calcium response (ΔF/F_0_) for response type ‘VORT 1-2 Peaks’ across time (x-axis). NR: no response. **f**, Max (top) and mean (bottom) peak amplitude of ΔF/F_0_ for type ‘BASE Ψ, VORT Ψ’ (n = 501 cells) displaying oscillatory calcium signaling during both time spans across the 6 cell lines in **d**. Paired two-tailed t-test. **g**, Resting membrane potential (Vm) of LN-229 (n = 13) and LN-308 cells (n = 10) measured by whole-cell patch-clamp before (CTRL) and after VORT treatment (10 µM) in matched single-cells (connected by grey lines). Paired two-tailed t-test. **h**, Representative single-cell current traces for each cell line (LN-229, LN-308) and condition (CTRL, VORT) corresponding to the voltage-clamp protocol (legend). **i**, Current-voltage characteristics (I-V curves) of LN-229 (n = 13) and LN-308 cells (n = 10) in **g**, before (CTRL) and after VORT treatment (10 µM) in matched single-cells. Standard error of the mean (SEM) shown as error bars. See *Methods* for description of summary statistics. **j**, Relative gene (panels) expression upon siRNA knockdown (columns) normalized to the *FLUC* negative control siRNA (n = 3 biological replicates; dots). Two-tailed t-test with adjusted *P*-values after Holm correction. Boxplots as in Fig. 1b. Source data

AP-1 activation and IEG expression are typically preceded by Ca^2+^-dependent signaling in neural lineage cells^44,50,52,53^. We, therefore, measured both extracellular Ca^2+^ influx as well as endoplasmic reticulum (ER) Ca^2+^ store release by high-throughput FLIPR assay (*n* = 17–18 drugs; Supplementary Table 2). Although none of the tested PCY-negative NADs and PCY-hit ONCDs triggered Ca^2+^ influx, five out of eight PCY-hit NADs, including anti-depressants vortioxetine, paroxetine and fluoxetine, elicited immediate and strong extracellular Ca^2+^ influx, not involving ER Ca^2+^ store release (Fig. 5e,f and Extended Data Fig. 7a). These results could be recapitulated in a PDC culture (P050.C; Extended Data Fig. 7b).

We delineated the single-cell Ca^2+^ dynamics elicited by vortioxetine, the most potent preclinical candidate, by live-cell Ca^2+^ imaging across four PDC cultures and two cell lines (n = 3,561 cells; Supplementary Video 1). Across all, vortioxetine robustly induced Ca^2+^ influx (Extended Data Fig. 7c), with the PDC cultures displaying baseline Ca^2+^ oscillations reminiscent of recent in vivo observations (Fig. 5g and Extended Data Fig. 7d,e)^21,23,24^. Vortioxetine increased the fraction of oscillating glioblastoma cells (Fig. 5g,h) and, for baseline oscillating cells, increased both their maximum peak amplitude (in 3/4 PDC lines) and mean peak amplitude (2/4 PDC lines) (Extended Data Fig. 7f). Electrophysiological characterization of vortioxetine response in LN-229 and LN-308 lines revealed LN-229-specific depolarization of the resting membrane potential (Extended Data Fig. 7g) and significant changes in the current-voltage characteristics (I-V curves) in both lines (Extended Data Fig. 7i,j). These results demonstrate that NADs and, in particular, vortioxetine rapidly alter glioblastoma neurophysiology preceding IEG/AP-1 upregulation.

Downstream of AP-1 upregulation, genome-wide mapping of transcriptional regulatory networks (PathwayNet)^54^ identified members of the AP-1 TF family to directly mediate BTG1/2 tumor suppressor gene expression (Fig. 5i). Furthermore, a strong correlation between the degree of induction of the COSTAR signature and the ex vivo anti-glioblastoma efficacy across NADs provided circumstantial evidence for a causal role of this GRN (R = 0.72, *P* = 1.4 × 10^−5^; Extended Data Fig. 6g). We, therefore, performed *BTG1/2* and *JUN* loss-of-function experiments (Fig. 5j,k and Extended Data Fig. 7j), after confirming knockdown efficiency (Extended Data Fig. 7j). Particularly *BTG1* knockdown accelerated LN-229 cell growth, measured by live-cell and end-point imaging (Fig. 5j,k and Supplementary Video 2). Furthermore, vortioxetine treatment after gene silencing revealed that *BTG1* knockdown attenuated vortioxetine’s anti-glioblastoma efficacy (Fig. 5k). Thus, vortioxetine engages an anti-proliferative program that includes AP-1/BTG-driven tumor suppression (Fig. 5l).

### Robust AP-1 induction across molecular regulatory layers

**Extended Data Fig. 8:**
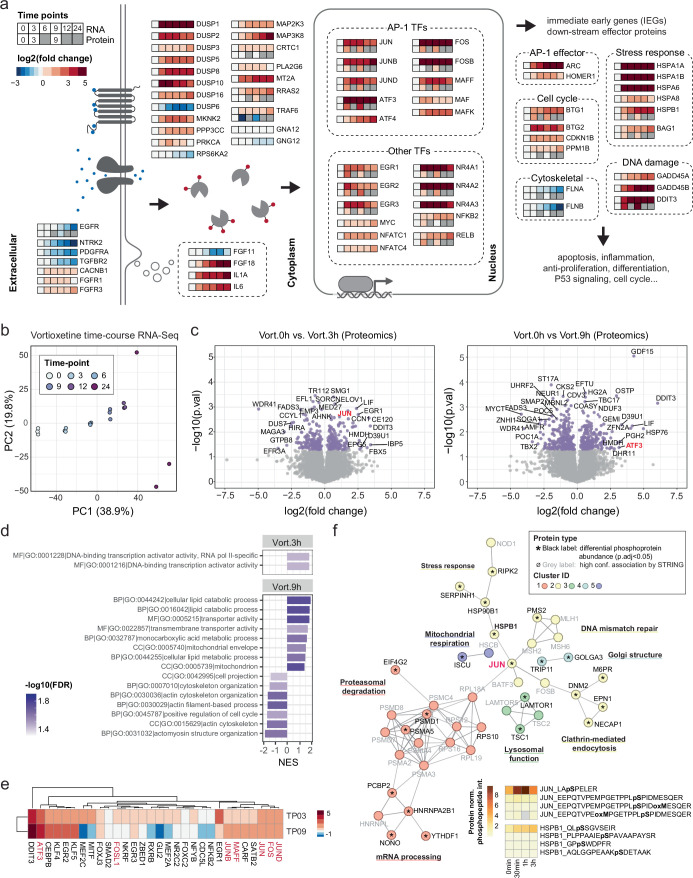
Vortioxetine induces an immediate and potent AP-1 response as measured by time-resolved transcriptomics, proteomics, and phosphoproteomics. **a**, Time-course visualization of AP-1 (PID) and MAPK (KEGG) pathway induction following Vortioxetine treatment (20 µM) in LN-229 cells measured by RNA-Seq (n = 6 time-points) and by proteomics (n = 3 time-points). n = 3 replicates/time-point. Genes selected for visualization are significantly differentially expressed by RNA-Seq at all time-points compared to the first time-point (0 h). Heatmap color scale represents log2(fold change) compared to the 0 h time-point. **b**, Principal component analysis (PCA) of replicate-averaged RNA-Seq counts following Vortioxetine treatment (20 µM) in LN-229 cells (n = 3 replicates/time-point) colored by time-point. **c**, Time-point comparisons (left, 3 h vs 0 h; right, 9 h vs 0 h) of proteomics measurements following Vortioxetine treatment (Vort, 20 µM; n = 3 replicates/condition) in LN-229 cells shown as volcano plots of log2(fold change) versus –log10(*P*-value). Proteins above a –log10(0.05 P-value) threshold are colored in purple. Two-tailed t-test. **d**, Gene Ontology (GO) gene set enrichment analysis of signed –log10(*P*-value) of comparisons in***c***. Bars represent the normalized enrichment score (NES) and colors indicate –log10(false discovery rate). **e**, Log2(fold change) in protein expression per time-point (rows; relative to 0 h) for the proteins (columns) contributing to enriched GO term “GO:0001216 DNA-binding transcription activator activity” in Extended Data Fig. 8d. AP-1 transcription factors are labeled in red. **f**, Connected protein-protein interaction network of differentially abundant phosphoproteins upon Vortioxetine treatment (20 µM; n = 3 replicates/condition) in LN-229 cells at any time-point. 22 out of 67 connected and significantly enriched phosphoproteins are shown (asterisks; black labels) with high confidence STRING protein interactions (grey labels). Cluster IDs (node colors) are based on the MCL algorithm with annotated biological pathways. Heatmap depicts protein abundance-normalized phosphopeptide (rows) intensities of JUN and HSPB1 across time-points (columns). Both genes are also significantly upregulated at the transcript level across all time-points. Source data

To profile the molecular response to vortioxetine, we performed deep transcriptomic, proteomic and phosphoproteomic profiling at 3–6 timepoints in LN-229 cells (Extended Data Fig. 8a–f). Rapid NH-2 terminal JUN phosphorylation after vortioxetine treatment was central to several differentially phosphorylated pathways, including the stress response pathway, mRNA processing and clathrin-mediated endocytosis (Extended Data Fig. 8f). Consistently, several AP-1 TFs, BTG1 and associated pathways, including MAPK signaling, ER stress and DNA damage response, were upregulated at both the RNA and protein level across all timepoints (Extended Data Fig. 8a,c,e). Conversely, vortioxetine treatment downregulated oncogenic RTKs, including EGFR, NTRK2 and PDGFRA (Extended Data Fig. 8a).

**Fig. 6:**
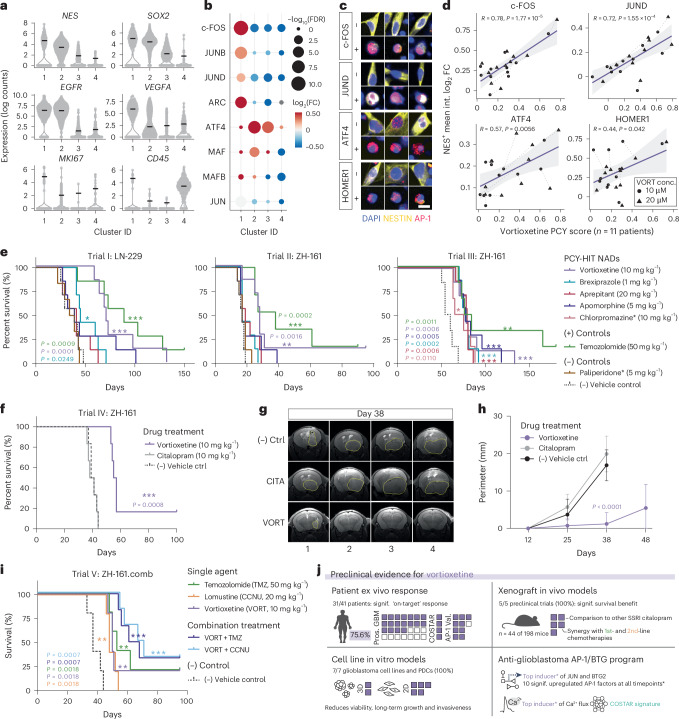
The anti-depressant vortioxetine confers significant survival benefit across preclinical trials and synergizes with standard-of-care glioblastoma treatments. **a**, scRNA-seq expression of select marker genes in patient sample P024. Cluster IDs are based upon UMAP clusters in Extended Data Fig. 9a. Black lines, median. **b**, Differentially expressed AP-1 TFs and effector gene ARC per scRNA-seq cluster in **a**, upon vortioxetine (VORT) treatment relative to DMSO. Circle sizes, −log_10_(adjusted *P* value); color scale, VORT-induced log_2_(fold change (FC)) compared to DMSO-treated cells per cluster. **c**, Example single-cell image crops from patient P040 of Nestin^+^ (yellow) cells after VORT treatment (+; 20 µM) and DMSO at 24 h stained with different AP-1 factors (red) and DAPI (blue). Scale bar, 15 µm. **d**, VORT ex vivo response (*x* axis; PCY score) versus AP-1 induction in Nestin^+^ glioblastoma cells by IF (*y* axis; log_2_(fold change) in mean intensity relative to DMSO) across patient samples (*n* = 11) at 24 h after VORT treatment (10 µM and 20 µM; VORT conc.). Pearson’s linear correlation coefficients and two-tailed *P* values are indicated. **e**, Survival analysis across three independent in vivo trials—Trial I: LN-229, Trial II: ZH-161 and Trial III: ZH-161—each with *n* = 6–7 tumor-bearing mice per treatment group and *n* = 7 treatments per trial. Doses are denoted in parentheses, and * indicates drugs used in a subset of the three trials. **f**, Survival analysis of in vivo Trial IV: ZH-161-iRFP720 tumor-bearing mice (*n* = 6 mice per treatment group). **g**, Representative MRI images of ZH-161-iRFP720 transplanted mice (columns; Trial IV; *n* = 4 mice) 38 d after tumor implantation (*n* = 3 drugs) with tumor perimeters indicated (yellow). **h**, Tumor perimeters of drug-treated mice in **g**, at multiple timepoints after tumor implantation by MRI. One-way ANOVA with adjusted *P* value from Tukey’s multiple comparisons test at day 38. **i**, Survival analysis of in vivo Trial V: ZH-161 tumor-bearing mice (*n* = 5–6 mice per group). **j**, Preclinical evidence for the top PCY-hit NAD VORT across modalities. AP-1 Val., AP-1 validation samples (*n* = 10 and *n* = 1 overlap with COSTAR); COSTAR, COSTAR validation samples (*n* = 4); Pros. GBM, prospective patient cohort (*n* = 27). *, among tested drugs and timepoints. **e**,**f**,**h**, Survival plotted as Kaplan–Meier curves and *P* values (colored by drug) calculated using log-rank (Mantel–Cox) test. Censored mice are denoted as tick marks. PCY-HIT, PCY-hit. Source data

**Extended Data Fig. 9:**
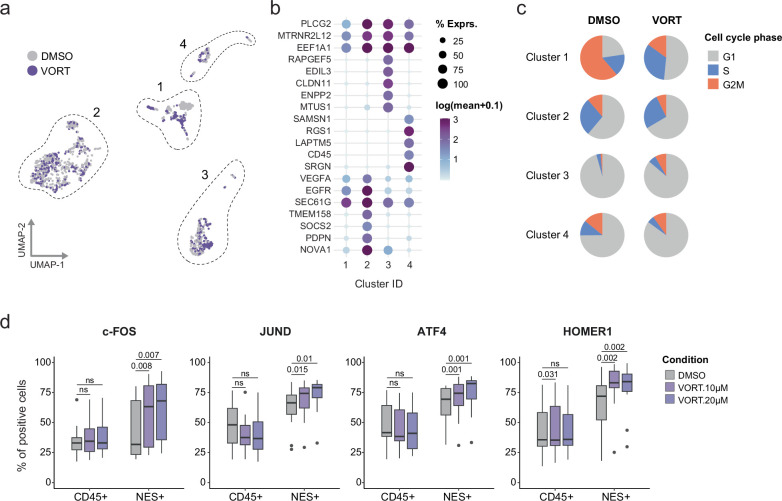
Single-cell RNA-Seq and immunofluorescence of Vortioxetine-treated glioblastoma patient cells. **a**, UMAP projection of 1736 single cells from patient sample P024 upon 3 h of treatment with Vortioxetine (VORT; n = 577 cells; purple; 20 µM) or DMSO vehicle control DMSO; n = 1159 cells; grey). **b**, Expression levels of the top five marker genes expressed in more than 10% of cells per scRNA-Seq cluster (columns) in Extended Data Fig. 9a. Circle sizes scale with the percent of cells within each cluster expressing each gene. Color scale represents log10(mean+0.1) expression. **c**, Cell cycle inference for each cluster in Extended Data Fig. 9b using Seurat version 4.3.0^80^. **d**, Percent of CD45+ immune or NES+ glioblastoma cells expressing AP-1 factors measured by immunofluorescence in glioblastoma patient samples (n = 11 patients) 24 h after DMSO control or Vortioxetine-treatment *ex vivo* (10 and 20 µM). Patient-matched paired two-tailed t-test (compared to DMSO control) with FDR-adjusted *P*-values. Boxplots show 25th–75th percentiles with a line at the median; whiskers extend to 1.5 times the interquartile range. Source data

Next, we performed scRNA-seq on patient cells after 3 h of ex vivo vortioxetine or DMSO treatment, revealing four cell clusters across the 1,736 single-cell transcriptomes (patient P024; Fig. 6a,b and Extended Data Fig. 9a,b). Clusters 1–3 represented glioblastoma cells expressing *Nestin*, *Ki67*, *EGFR* and *VEGFA*, with cluster 1 showing the most aggressive signature and highest inferred fraction of cells in the G2M cell cycle phase (Extended Data Fig. 9c). Analyzing the transcriptional response to vortioxetine treatment revealed a reduction of inferred G2M phase cells (Extended Data Fig. 9c) and confirmed glioblastoma-specific induction of AP-1 TFs and effector genes in patient cells (Fig. 6b).

Profiling the vortioxetine response at the AP-1 protein level across patient samples revealed that the patient response heterogeneity correlated with the degree of AP-1 upregulation (across c-FOS, JUND, ATF4 and the AP-1 effector HOMER1; Fig. 6c,d). Consistent with the scRNA-seq analysis, AP-1 induction was specific for glioblastoma cells, whereas immune cells did not exhibit AP-1 induction and showed lower baseline AP-1 expression (Extended Data Fig. 9d). Thus, this single-cell analysis across patients identified AP-1 induction as a predictor of vortioxetine efficacy and validated the glioblastoma-specific therapeutic convergence of NADs on AP-1/BTG-driven tumor suppression across modalities and patient heterogeneity.

### Anti-depressant vortioxetine is the strongest preclinical candidate

**Extended Data Fig. 10:**
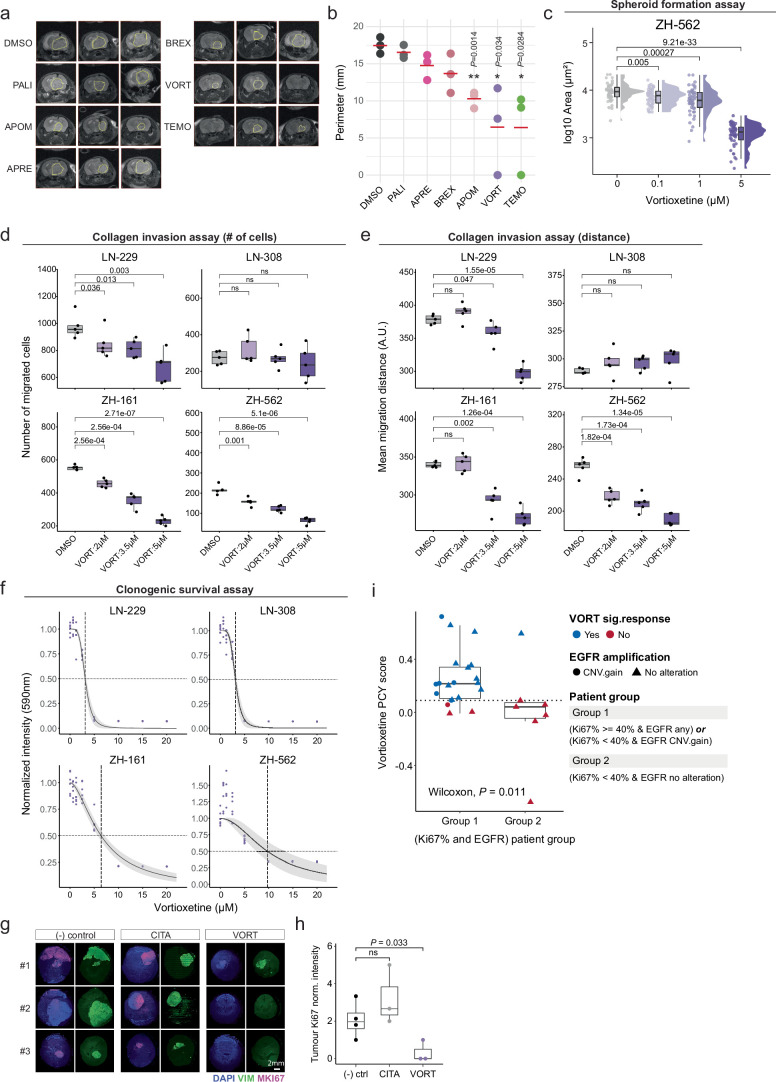
Vortioxetine reduces tumor burden in vivo independent of serotonin modulation and affects tumor invasiveness and long term growth. **a**, Representative MRI images of three ZH-161 transplanted mice (columns) after 15 days of drug treatment (Trial II; n = 7 drugs). Tumor perimeters indicated in yellow. **b**, Quantification of tumor perimeters corresponding to ***a***. Dots: individual mice per drug (columns); Red lines: mean values. Two-tailed t-test. **c**, Spheroid formation analyzed by the 2D-projected area of the ZH-562 line measured after 12 days of Vortioxetine treatment (0.1-5 µM; n = 45-47 wells/condition). Data is shown as a boxplot, individual data points, and histogram. **d**, Number of migrated cells in a collagen-based spheroid invasion assay after 36 h of Vortioxetine treatment (2, 3.5, 5 µM) across four glioblastoma cell lines; LN-229 (n = 560-1125 cells/well), LN-308 (n = 137-426 cells/well), ZH-161 (n = 200-574 cells/well), ZH-562 (n = 38-253 cells/well). **e**, Mean cell migration distance per condition (n = 5 replicate wells) for ***d***. **c-e**, One-tailed t-test with adjusted *P*-values after Holm correction. **f**, Clonogenic survival measured by a resazurin-based cell viability assay after 11-13 days of Vortioxetine treatment (7 concentrations; 0.625-20 µM, n = 6 replicate wells/concentration) across four glioblastoma cell lines; LN-229 (n = 50 cells/well), LN-308 (n = 300 cells/well), ZH-161 (n = 500 cells/well), and ZH-562 (n = 500 cells/well). Dose-response fitted with a two-parameter log-logistic distribution with 95% confidence intervals (grey) and ED50 (dashed lines). **g**, Representative immunohistochemistry images of brain sections (n = 3 mice/treatment group) stained with human-specific Ki67 and Vimentin (VIM). **h**, Ki67 tumor intensity normalized to background with n = 3-4 mice (dots) analyzed per group. Two-tailed t-test comparing CITA and VORT treatment to (-) ctrl. **i**, Vortioxetine *ex vivo* PCY score (n = 27 patients; prospective cohort) stratified by Ki67 levels and *EGFR* CNV alterations. Group 2 patients with low Ki67 levels and an absence of *EGFR* CNV alterations (n = 7/27; 26%) were significantly less likely to respond to Vortioxetine *ex vivo* compared to Group 1 (Wilcoxon test; *P* = 0.011). Among the clinical/genetic parameters in Fig. 2d, e, Ki67 and *EGFR* alterations were the most predictive two parameters based on a regression subset selection for *ex vivo* Vortioxetine response. **c, d, e, i, h**, Boxplots as in Fig. 1b. Source data

Finally, to evaluate the in vivo anti-glioblastoma efficacy of our top NADs, we tested PCY-hit NADs spanning different drug classes in two distinct orthotopic human xenograft glioblastoma mouse models (LN-229 and ZH-161) across four independent preclinical trials (Trials I–IV; Fig. 6e–h and Extended Data Fig. 10a,b). Standard-of-care TMZ was included as positive control, and PCY-negative NADs paliperidone or citalopram and vehicle were negative controls. Treatment doses were determined a priori based on literature and clinical evidence.

Vortioxetine was consistently the most effective PCY-hit NAD in vivo (in 4/4 trials), showing significant survival benefit, similar to TMZ (Fig. 6e,f). Furthermore, vortioxetine treatment significantly reduced tumor size in vivo measured by magnetic resonance imaging (MRI) of ZH-161 transplanted mice after 15 d (Trial II; Extended Data Fig. 10a,b), and vortioxetine displayed multifaceted anti-tumor effects in vitro: it reduced glioblastoma growth, invasiveness and clonogenic survival across 2D and 3D glioblastoma cell lines (Extended Data Fig. 10c–f). Brexpiprazole was the second-best PCY-hit NAD in vivo (in 2/3 trials), and other NADs conferred significant survival benefit in single trials (Fig. 6e). Consistent with our ex vivo PCY results, the negative controls paliperidone (in 2/2 trials; Fig. 6e) and citalopram (single trial; Fig. 6f) showed no survival benefit. The confirmed lack of efficacy of anti-depressant citalopram, in particular, highlights that serotonin modulation alone does not confer anti-glioblastoma efficacy (Trial IV; Fig. 6f). In this direct dose comparison, only vortioxetine lowered Ki67 levels in situ, reduced tumor burden and increased survival (Fig. 6f–h and Extended Data Fig. 10g,h).

The striking consistency of our patient ex vivo and mouse in vivo results demonstrates strong translatability of PCY-based NAD discovery and confirms vortioxetine as the most promising clinical candidate. To prepare its clinical translation, we further tested the combination of vortioxetine with either first-line or second-line standard-of-care chemotherapies for glioblastoma, TMZ and lomustine (CCNU) in vivo (Trial V: ZH-161; Fig. 6i). All three single agents significantly prolonged survival, with vortioxetine results now confirmed in five out of five in vivo trials (Fig. 6e,f,i). Compared to TMZ or CCNU single agents, the combination of vortioxetine with either drug provided a further median survival increase of 20–30%, with four out of 12 mice (25%) displaying long-term survival (Fig. 6i). The added survival benefit conferred by these neuro/chemo combination therapies supports the mechanistic complementarity of neuroactive AP-1/BTG tumor suppression with the current standard of care targeting genome integrity. Lastly, we identified that patients with low Ki67 levels and absence of *EGFR* alterations were the least likely to benefit from vortioxetine treatment ex vivo (Extended Data Fig. 10i), offering a patient stratification strategy for this strong preclinical candidate (Fig. 6j).

## Discussion

Here we present a therapeutic single-cell map across patient samples that reveals the molecular NAD landscape of glioblastoma. Driving this discovery is the high-throughput functional evaluation of glioblastoma tissue shortly after surgery across heterogeneous patient cohorts by PCY. In line with the prior successful use of PCY to guide patient treatment for blood cancers and growing community efforts in functional precision oncology^32,33,35,55,56^, our results indicate the feasibility of using PCY as a drug discovery and personalized treatment selection platform for hard-to-treat solid tumors.

Our prospective ex vivo testing of repurposable drugs expands the investigation of NADs^57–59^, patient-derived explants^27,38,39,60–62^ and molecular predictors of response to accelerate clinical translation of therapeutic candidates for glioblastoma. Near real-time drug testing further addresses limitations of retrospective cohort studies examining coincidental NAD use, which can be confounded by the time of prescription and grouping of multiple drugs. Expansion to larger cohorts and consideration of other important aspects of glioblastoma etiology, including the metabolic state^30,63^, spatial tumor organization^64,65^ and the neuron–glioma interface^18,21–23,25^, will further consolidate our understanding of patient heterogeneity and treatment response.

Despite many possible neural vulnerabilities, our interpretable machine learning (COSTAR) identified a convergent drug–target connectivity signature predictive of anti-glioblastoma efficacy across drugs. COSTAR effectively applies Occam’s razor to the collective biochemical drug–protein–protein interaction network, offering a conceptual framework likely applicable to other cancers and drug discovery efforts. Using COSTAR, deep multi-omic profiling and functional genetics, we uncovered NAD-specific convergence through AP-1 activity on BTG-mediated tumor suppression. However, the chemical properties leading to AP-1 upregulation remain to be identified, and polypharmacological mechanisms likely contribute to the integrated effect of individual NADs.

Previous studies demonstrated the role of neuronal input in regulating glioblastoma growth at the brain–tumor interface, highlighting the influence of the TME in modulating the neural behavior of the tumor^18,21–23,25^. In the present study, we uncovered a tumor-intrinsic neural vulnerability in glioblastoma, offering a therapeutic window that enables direct targeting of tumor neurophysiology independent of neuronal input. In cancers, AP-1 factors were initially discovered as oncogenes, although an increasing number of studies report context-dependent anti-oncogenic functions of AP-1 factors. In contrast, for neurons and other neural lineage cells, IEG expression of AP-1 factors is typically a hallmark of neural activity or insult^42,44–46^.

In the context of glioblastoma cells, we now report that diverse NADs—particularly the anti-depressant vortioxetine—target this neural activity-like signaling, triggering a strong neurophysiological and transcriptional response that leads to rapid glioblastoma cell death. Vortioxetine’s potency was orthogonally demonstrated across modalities, with an on-target ex vivo efficacy observed in 75% of patients (Fig. 6j). Moving forward, vortioxetine in combination with standard-of-care chemotherapeutics should be tested in controlled clinical trials, potentially guided by molecular or functional patient stratification. Treating glioblastoma tailored to the cellular history and lineage of the cancer, in addition to its unstably transformed state, offers hope for this devastating disease.

## Methods

### Patient sample processing

#### Ethics statement and patient cohort

Adult patients with IDH-wildtype glioblastoma and CNS World Health Organization (WHO) grade 4 according to the 2021 WHO classification of CNS tumors treated either at the University Hospital of Zurich or the Cantonal Hospital St. Gallen provided informed consent to take part in the study with approval by the institutional review board (ethical approval number KEK-StV-Nr.19/08; BASEC numbers 2019-02027 and 2021-00652). There was no limit on tumor size for the human samples included in the study and no selection bias of the enrolled patients. Clinical characteristics of the prospective and retrospective patient cohorts, including clinical parameters, experiment inclusion, sex, age and genetics summary, can be found in Supplementary Table 1 and Supplementary Data 1. The prospective cohort consists of patients where fresh tissue was processed directly within 4 h after surgery (*n* = 27 patients for drug screening and an additional *n* = 17 patients for validation experiments). For PFS analysis of the prospective cohort, only patients with newly diagnosed glioblastoma who received radiotherapy and TMZ chemotherapy were included. The retrospective cohort (*n* = 18 patients) consists of patients for whom bio-banked tissue was available and who received maintenance TMZ, with OS documented as a clinical endpoint. Retrospective samples were selected to cover a broad spectrum of PFS outcomes and were further selected based on quality control measures, including cell viability, cell number and the amount of debris present in the sample.

#### Patient sample dissociation for ex vivo drug screening

Surgically removed tissue samples were first washed with PBS and cut using single-use sterile scalpels. Subsequent dissociation was performed in reduced serum media (DMEM media, no. 41966, with 2% FBS no. 10270106, 1% pen–strep no. 15140122 and 25 mM HEPES no. 15630056, all from Gibco) supplemented with Collagenase IV (1 mg ml^−1^) and DNaseI (0.1 mg ml^−1^) using a gentle MACS Octo Dissociator (Miltenyi Biotec, 130-096-427) for maximally 40 min. Homogenates were filtered through a 70-µm cell strainer (Sigma-Aldrich, CLS431751) and washed once with PBS containing 2 mM EDTA. Myelin and debris removal was performed by a gradient centrifugation of the cell suspension in a 7:3 mix of PBS:Percoll (Sigma-Aldrich, P4937), with an additional PBS wash. In case the cell pellet visibly contained a notable portion of red blood cells (RBCs), RBC lysis was performed with 1× RBC lysis buffer (BioLegend, 420301) at room temperature for 10 min before the PBS wash. Subsequently, cells were resuspended in reduced serum media, filtered once more through a 70-µm cell strainer and counted using a Countess II Automated Cell Counter (Invitrogen). In case sufficient cell numbers remained after cell seeding for ex vivo drug testing, cells were cryopreserved in 10% DMSO-containing cryopreservation media and/or cultured in DMEM media supplemented with 10% FBS, 1% pen−strep and 25 mM HEPES to obtain PDCs shortly maintained for a few passages as adherent cultures.

### Cell culture

The adherent human glioblastoma cell lines LN-229 (American Type Culture Collection, CRL-2611, directly purchased from the vendor), LN-308 (obtained from the University Hospital of Zurich) and PDCs (patient IDs denoted with a ‘.C’) were cultured in standard serum media (DMEM media, no. 41966, with 10% FBS no. 10270106, 1% pen–strep no. 15140122 and 25 mM HEPES no. 15630056, all from Gibco). Adherent cell lines and PDCs were passaged using trypsin-EDTA (0.25%, Gibco, 25200056), with PDCs shortly maintained for a few passages after surgical dissociation. The spheroid human glioblastoma-initiating cell lines ZH-161 and ZH-562 were generated at the University Hospital of Zurich and cultured in Neurobasal (NB) medium (Gibco, 21103049) supplemented with B27 (Gibco, 17504044), 20 ng ml^−1^ b-FGF (PeproTech, AF-100-18B), 20 ng ml^−1^ EGF (PeproTech, AF-100-15) and 2 mM L-glutamine (Gibco, 25030081). Suspension spheroid cultures were passaged using Accutase (STEMCELL Technologies, 07920). Cell lines LN-308, ZH-161 and ZH-562 were authenticated at the Leibniz Institute DSMZ by short tandem repeat (STR) DNA analysis, whereas LN-229 was not authenticated as it was bought directly from the vendor. The LN-229 line is derived from a female patient, and LN-308, ZH-161 and ZH-562 are derived from male patients. LN-229 and LN-308 lines have methylated *MGMT* promoters. The LN-229 line is commercially available, and other glioblastoma cell lines/PDCs are obtainable from either the University Hospital of Zurich or the Snijder laboratory with the exception of the P022.C line that was not able to be expanded beyond five passages. All cell cultures were maintained at 37 °C, 5% CO_2_ in a humidified incubator.

### PCY (drug testing, immunocytochemistry, confocal microscopy and image analysis)

The PCY method refers to high-content image-based ex vivo drug testing, including the following steps of cell seeding, drug testing, immunocytochemistry, confocal microscopy, image analysis and PCY score calculation for each tested drug^32,35^.

#### Cell seeding and drug testing

Freshly dissociated cells were seeded into CellCarrier 384 Ultra microplates (PerkinElmer, 6057300) typically within 4 h of surgery with 0.5–1.5 × 10^4^ cells per well. For cultured glioblastoma cell lines and PDCs, trypsinized (adherent cultures) or accutase-treated (spheroid cultures) cells were seeded at 0.5–2.5 × 10^3^ cells per well in 384-well plates. Before cell seeding, drugs were re-suspended as 5 mM stock solutions and dispensed into 384-well plates using an Echo 550 liquid handler (Labcyte) in a randomized plate layout to control for plate effects. Detailed information regarding drugs used in this study can be found in Supplementary Table 2. Different drug libraries included glioblastoma drugs (GSDs, *n* = 3 drugs), ONCDs (*n* = 65 drugs) and NADs (*n* = 67 drugs). The NAD library was based on purchasable drugs from the vendor (Sigma-Aldrich) of *n* = 119 CNS marketed drugs cited in Wager et al.^66^ and a curated list of *n* = 35 FDA-approved drugs for CNS indications between 2010 and 2018 after Wager et al.^66^ was published. All NADs were tested at 20 μM, and, for select NADs, a concentration range of 0.1–100 μM was tested (Extended Data Fig. 3a–d). GSDs were tested at the following concentrations: TMZ (first-line glioblastoma chemotherapy; 50, 100, 250 and 500 µM) and CCNU and carmustine (BCNU) (second-line glioblastoma chemotherapies; 10, 50, 100 and 250 µM). All ONCDs were tested at 10 μM concentrations. Drug plates included the following number of replicate wells per drug/concentration: GSD plate, drug, *n* = 3 wells, DMSO, *n* = 16 wells; NAD plate, drug, *n* = 4 wells, DMSO, *n* = 16–24 wells; ONCD plate, drug, *n* = 4 wells, DMSO, *n* = 16 wells. Cells were incubated for 48 h with drugs in reduced serum media at 37 °C, 5% CO_2_ before proceeding to cell fixation.

#### Immunocytochemistry

Cells were fixed with 4% paraformaldehyde (PFA) (Sigma-Aldrich, F8775) in PBS and blocked in 5% FBS and 0.1% Triton containing PBS. For characterization of cellular composition, cells were stained overnight at 4 °C in blocking solution with the following antibodies and dilutions: Alexa Fluor 488 anti-S100 beta (1:1,000, Abcam, ab196442, clone EP1576Y), PE anti-Nestin (1:150, BioLegend, 656806, clone 10C2), Alexa Fluor 488 anti-CD3 (1:300, BioLegend, 300415, clone UCHT1), Alexa Fluor 647 anti-CD45 (1:300, BioLegend, 368538, clone 2D1) and DAPI (1:1,000, BioLegend, 422801, stock solution 10 mg ml^−1^). Due to the temporary manufacturer discontinuation of the Alexa Fluor 488 anti-S100 beta antibody, from patient sample P030 and onwards, samples were stained with either a self-conjugated Alexa Fluor 488 anti-S100 beta antibody, where Alexa Fluor 488 NHS Ester (Thermo Fisher Scientific, A20000) was conjugated to the anti-S100 beta antibody (Abcam, ab215989, clone EP1576Y), or the following antibody panel where the 488 and 555 channel markers were swapped: Alexa Fluor 488 anti-Nestin (1:150, BioLegend, 656812, clone 10C2), Alexa Fluor 555 anti-S100 beta (1:1,000, Abcam, ab274881, clone EP1576Y), PE anti-CD3 (1:300, BioLegend, 300441, clone UCHT1) and Alexa Fluor 647 anti-CD45 (1:300, BioLegend, 368538, clone 2D1).

Other conjugated antibodies used included Alexa Fluor 647 anti-tubulin beta 3 (1:1,000, BioLegend, 657406, clone AA10); Alexa Fluor 488 anti-vimentin (1:500, BioLegend, 677809, clone O91D3); Alexa Fluor 555 anti-cleaved caspase-3 (1:500, Cell Signaling Technology, 9604S); Alexa Fluor 546 anti-HOMER (1:300, Santa Cruz Biotechnology, sc-17842 AF546, clone D-3); PE anti-CFOS (1:300, Cell Signaling Technology, 14609S, clone 9F6); FITC anti-ATF4 (1:300, Abcam, ab225332); Alexa Fluor 488 anti-JUND (1:300, Santa Cruz Biotechnology, sc-271938 AF488, clone D-9); and Alexa Fluor 594 anti-CD45 (1:300, BioLegend, 368520, clone 2D1). Other unconjugated antibodies used included anti-Connexin43 (1:500, Cell Signaling Technology, 83649T); anti-EGFR (1:300, Abcam, ab98133); anti-Nestin (1:150, BioLegend, 656802, clone 10C2); anti-S100 beta antibody (1:300, Abcam, ab215989, clone EP1576Y); and anti-Ki67 (1:300, Cell Signaling Technology, 9129S, clone D3B5). For unconjugated primary antibodies, the following secondary antibodies were used: donkey anti-sheep IgG (H + L) cross-adsorbed secondary antibody, Alexa Fluor 488 (Thermo Fisher Scientific, A11015); goat anti-mouse IgG (H + L) highly cross-adsorbed secondary antibody, Alexa Fluor Plus 555 (Thermo Fisher Scientific, A32727); and goat anti-rabbit IgG (H + L) highly cross-adsorbed secondary antibody, Alexa Fluor Plus 647 (Thermo Fisher Scientific, A32733). All secondary antibodies were used at 1:500 dilution.

#### Confocal imaging and image analysis

Imaging of 384-well plates was performed with an Opera Phenix automated spinning-disk confocal microscope (PerkinElmer, HH14000000) at ×20 magnification for all assays with the exception of spheroid cell lines (ZH-161 and ZH-562) imaged at ×10 magnification. Select images were imaged at ×40 for visualization. Single cells were segmented based on their nuclei (DAPI channel) using open-source CellProfiler 2.2.0, and nuclear expansion was performed to assess cytoplasmic features, including marker expression. Downstream image analysis was performed with MATLAB R2019a–R2020a. Fractions of marker-positive cells for each sample and drug condition were based on local background-corrected intensity histograms across the whole drug plate. In patient samples, marker-positive populations were defined as follows: glioblastoma cells ((Nestin^+^ or S100B^+^) and CD45^−^), immune cells (CD45^+^ and S100B^−^Nestin^−^) and other marker-negative cells (S100B^−^Nestin^−^CD45^−^). Marker-positive fractions were averaged across each well/drug.

#### PCY score calculation

The PCY score quantifies the drug-induced relative reduction of any marker-defined cell population by measuring the change of a defined target population upon drug treatment compared to DMSO vehicle control. In patient samples, the PCY score is calculated based on the fraction of ((Nestin^+^ or S100B^+^) and CD45^−^ cells) out of all cells. In PDC lines, the score is based on (Nestin^+^) cells out of all cells. By all cells, we refer to any detected cell with a DAPI^+^ nucleus. PCY scores are averaged across technical replicates for each drug or control condition.

$${\rm{PCY}}\; {\rm{score}}=1-\{[{{\rm{TP}}_{\rm{DRUG}}}]\div[{{\rm{TP}}_{\rm{DMSO}}}]\}$$

where TP_DRUG_ = fraction of the target population in a given DRUG condition of all cells and TP_DMSO_ = fraction of the target population in the DMSO control condition of all cells.

A positive PCY score of 1 represents the strongest possible ‘on-target’ response; a PCY score of 0 indicates no effect/equal cytotoxicity; and a negative PCY score indicates higher toxicity to other cell populations other than the defined target population. In cases where a target population is not defined, drug response and cell viability are measured as total cell number reduction in LN-229 and LN-308 lines and a reduction of 2D projected total spheroid area in ZH-161 and ZH-562 lines.

#### Deep learning of apoptotic cell morphologies

To generate a training dataset, cleaved CASP3^+/−^ cells identified by IF and CellProfiler-based image analysis (*n* = 6 patient samples) were cropped as five-channel 50 × 50 pixel images around the nuclear centroid of each cell. In total, 6,072 single-cell image crops were manually curated and labeled as two classes (CASP3^+/−^) based on their cleaved CASP3 staining. A convolutional neural network (CNN) with a modified AlexNet architecture^67^ with the image input size set as 50 × 50 × 2 (two-channel bright-field (BF) and DAPI classifier) and the number of output classes set to 2 (CASP3^+/−^) was then trained on this manually curated image dataset (*n* = 6,072 single-cell images; split by a 8:2 ratio into training and test data, respectively). CNN training included use of the Adam optimizer, with a mini-batch size of 64 and a maximum number of 30 epochs. The initial learning rate was set to 0.01 with a piece-wise learning rate schedule and a drop factor of 0.1 every 10 epochs. Network performance on a manually curated test image dataset (*n* = 1,214 single-cell crops) is shown as a confusion matrix in Extended Data Fig. 1j. All DAPI^+^ nuclei detected in patient samples were retrospectively classified by this apoptotic classifier CNN based on the BF and DAPI channels to quantify apoptotic fractions across the prospective patient cohort, marker-based subpopulations and drug conditions. Cells were classified as apoptotic (CASP3^+^) based on a CNN confidence threshold of 87%, close to the true-positive rate of the classifier.

#### Demonstration of PCY score robustness to apoptotic cells

We performed ex vivo NAD (*n* = 67 drugs) screens in two patient samples (P048 and P049) by staining for cleaved CASP3. The drug response (Extended Data Fig. 2i–k) shows excellent reproducibility, both when comparing the original PCY scores with the PCY scores obtained after excluding CASP3^+^ cells by IF as well as when comparing the PCY scores after excluding CASP3^+^ cells defined either by IF or by the CNN apoptotic classifier. We also re-calculated the PCY scores by excluding the CNN-classified apoptotic cells measured across all 27 patient samples and 67 NADs and compared them to the original PCY scores reported in the manuscript (Extended Data Fig. 2k). The drug response correlation with or without the inclusion of apoptotic cells was 0.988, demonstrating that the PCY score is highly robust to the presence of apoptotic cells (Extended Data Fig. 2k) and can be expected to be equally robust to other forms of cell death.

### Targeted next-generation sequencing (Oncomine Comprehensive Assay)

Formalin-fixed paraffin-embedded (FFPE) tissue blocks from patient-matched samples collected from the University Hospital of Zurich were used to determine genetic alterations. Tumor areas were marked on the hematoxylin and eosin (H&E) slide, and relative tumor content was estimated by a trained pathologist. One to three core cylinders (0.6-mm diameter) from the FFPE blocks (tumor areas) were used for DNA and RNA isolation. DNA was isolated with a Maxwell 16 FFPE Tissue LEV DNA Purification Kit (Promega, AS1130). DNA concentration was determined using a Qubit dsDNA HS Assay Kit. RNA was extracted with a Maxwell 16 FFPE Tissue LEV RNA Purification Kit (Promega, AS1260) after pre-treatment with DNase1 for 15 min at room temperature. Library preparation with 20 ng of DNA or RNA input was conducted using Oncomine Comprehensive Assay version 3. Adaptor/barcode ligation, purification and equilibration were automated with Tecan Liquid Handler (EVO-100). Next-generation sequencing (NGS) libraries were templated using Ion Chef and sequenced on an S5 (Thermo Fisher Scientific), and data were analyzed using Ion Reporter software 5.14 with Applied Filter Chain: Oncomine Variants (5.14) settings and Annotation Set: Oncomine Comprehensive Assay version 3 Annotations version 1.4. For NGS data analysis, Torrent Suite software (Ion Reporter) was used, enabling detection of small nucleic variants (SNVs), copy number variations (CNVs), gene fusions and indels from 161 unique cancer driver genes.

Detected sequence variants were evaluated for their pathogenicity based on previous literature and the ClinVar database^68^. Gene alterations described as (likely) benign were not included in the results. Non-pathogenic mutations harboring a minor allele frequency higher than 0.01 were not selected. The Default Fusion View parameter was selected. For the CNV confidence range, the default filter was used to detect gains and losses using a 5% confidence interval (CI) for minimum ploidy gain over the expected value and 95% CI for minimum ploidy loss under the expected value. CNV low-confidence range was defined for gain by copy number from 4 to 6 (minimum CNV CI 5%: 2.9) and loss from 0.5 to 1 (maximum CNV CI 95%: 2.43). High-confidence range was defined by gain up to 6 copy number (minimum CNV CI 5%: 4.54) and loss below 0.5 copy number (maximum CNV CI 95%: 1.37). The 5% and 95% CIs of all selected loss and gain are available in Supplementary Data 1. The minimum number of tiles required was 8. Results are reported as detected copy number.

### scRNA-seq and re-analysis of other published datasets

Cryopreserved patient samples were thawed and used for scRNA-seq. Viability markers SYTOX Blue (1 μM, Thermo Fisher Scientific, S11348) and DRAQ5 (1 μM, BioLegend, 424101) were added to the cell suspension at least 15 min before sorting. Fluorescence-activated cell sorting (FACS) gates were set based on CD45 (Alexa Fluor 594 anti-CD45, 1:20, BioLegend, 368520, clone 2D1) and SYTOX Blue/DRAQ5 intensities to sort viable CD45^+^ and CD45^−^ populations (Extended Data Fig. 1a) into DNA LoBind Eppendorf tubes (VWR, 525-0130) using the BD FACSAria Fusion Cell Sorter and FlowJo 10.4.2 software. CD45^−^ and CD45^+^ cells were mixed at 2:1 to 10:1 ratios depending on availability to enrich for glioblastoma cells. Single-cell transcriptomes from four patient samples (P007, P011, P012 and P013), part of the prospective cohort, are referred to as ‘Lee et al.; this study’. For patient sample P024 that was used to measure the effect of vortioxetine drug treatment, cells sorted by FACS were incubated for 3 h with or without 20 µM vortioxetine before proceeding to library preparation. Libraries were generated using Chromium Next GEM Single Cell 3′ version 3.0 and version 3.1 kits (10x Genomics) and sequenced on a NovaSeq 6000 (Illumina). Read alignment to the GRCh38 human reference genome, generation of feature–barcode matrices and aggregation of multiple samples were performed using the Cell Ranger analysis pipeline (10x Genomics, versions 3.0.1 and 6.1.1). Four patient samples were processed in November 2019 with the earlier version of 10x Genomics library prep kits and Cell Ranger analysis pipeline, whereas the later sample (P024) was processed in September 2021.

#### Analysis of the cohort-matched in-house scRNA-seq dataset

Quality control for the in-house dataset (Lee et al.) was performed by analyzing only high-quality cells with less than 10% of mitochondrial transcripts and genes that had at least a count of 2 in at least three cells. For the Lee et al. dataset, an expression threshold of log_2_(count+1) > 3 was applied to consider a gene expressed in a given cell. Uniform manifold approximation and projection (UMAP) clusters in Extended Data Fig. 1c are based on Leiden community detection, and cell types are assigned by marker expression. Glioblastoma clusters are numbered in descending order based on cluster-averaged expression of the Gene Ontology term ‘stem cell differentiation’ (GO:0048863).

#### Re-analysis of other published scRNA-seq datasets

To analyze additional glioblastoma patient cohorts by scRNA-seq, we used two published datasets: Neftel et al.^4^ and Yu et al.^40^. For Neftel et al., we removed cells with fewer than 2^9^ detected genes and/or more than 15% of mitochondrial transcripts. For Yu et al. the data were already pre-filtered, but patient samples (7–9 and 14–15) that did not correspond to glioblastoma (grade IV astrocytomas) were not included. For both datasets, only genes that had at a count of 2 in at least two cells were included in the analysis. For the Neftel et al. and Yu et al. datasets, expression thresholds of log_2_(count+1) over 5 and 3, respectively, were applied to consider a gene expressed in a given cell. For all three scRNA-seq datasets, only patient samples with more than 50 positive cells for a given gene were considered in Fig. 1b and Extended Data Fig. 1d.

#### Inferred CNA analysis

CNAs were inferred using the ‘infercnv’ R package (version 1.18.0), using the same cell type definition in Fig. 1b and expression threshold as described above, sampling up to 70 cells per patient and cell type. ‘infercnv’ was run on the sampled cells with default settings with *CD45*^+^ immune cells across all patients set as the reference cell type. A cell was considered to have a detectable CNA if the mean ‘modified expression’ across all genes on each respective chromosome was either above a threshold of 1.1 for chromosome 7 (amplification) or below 0.9 for chromosome 10 (loss). Only patient samples that had detectable CNAs for their respective chromosomes in at least 5% of cells (combined across ‘*Nestin*^+^ or *S100B*^+^’ and ‘other’ cells) were included in the analysis presented in Fig. 1c.

#### Cell-type-specific enrichment analysis of gene modules enriched in ‘other’ cells

To determine putative cell types represented in *Nestin*^−^*S100B*^−^*CD45*^−^ cells (‘other’) by scRNA-seq, we analyzed the log_2_(fold change) of ‘other’ enriched genes compared to glioblastoma cells. First, an aggregated average ‘metacell’ for each patient and subpopulation (either ‘other’ or glioblastoma cells) was created by summing the counts across each [patient-subpopulation] and dividing this by the corresponding number of cells. Next, considering only genes where the aggregate-averaged expression is above 1 in at least one ‘metacell’ type, we calculated the log_2_(fold change) of [‘other’ metacell] / [glioblastoma metacell] per gene and per patient. Manhattan distance-based clustering of the top 10 log_2_(fold change) of ‘other’ enriched genes per patient is visualized in Extended Data Fig. 1g. Dendrogram tree cutting of ‘other’ enriched genes yielded gene modules that were analyzed by WebCSEA^69^ to determine most likely cell types represented by the respective gene modules. The top seven most likely cell types representing each ‘other’ gene module ranked by the lowest combined *P* values are shown in Extended Data Fig. 1h.

#### Neural specificity and patient specificity score analysis

Neural specificity and patient specificity scores for each gene were defined as follows. Using the in-house dataset, we identified putative cell types by unsupervised clustering using Monocle^70^ and annotated the clusters as being either immune cells or neural cells based on known marker genes. DESingle^71^ analysis resulted in 11,571 neural-specific and 1,157 immune-specific genes (log_2_FC > 0.5). Using these cell-type-specific gene sets, we calculated an immune score and a neural score for each cell using singscore, and we classified every cell in the additional datasets as either neural or immune based on a linear combination of both scores. The ‘*neural specificity score’* is defined as follows: [*neural specificity = fraction of neural cells expressing gene – fraction of immune cells expressing gene*] where expression of a given gene in a cell is defined as having any non-zero count. This score ranges from −1 (gene is expressed in all immune cells and no neural cells) to +1 (gene is expressed in all neural cells and no immune cells). For genes with low expression, this score will be close to 0, reflecting the fact that clear statements cannot be made about cell type specificity for these genes. To assess the variation of gene expression across patients, we defined a ‘*patient specificity score’* as follows. First, for every gene *gi* and every patient *pj*, we calculated a cell type composition independent fraction of cells expressing gene *gi* as [*Fraction_expressing_ij = fraction_expressing_immune_ij + fraction_expressing_neural_ij*]. We then defined patient specificity as the median absolute deviation (MAD) of fraction_expressing across all patients, thus defining [*Patient_specificity_i = mad(Fraction_expressing_i,:)*].

### siRNA knockdown and quantitative real-time PCR

All siRNAs used in the study were part of the MISSION esiRNA library (Sigma-Aldrich, Euphoria Biotech; Supplementary Table 4) and ordered as custom gene arrays (esiOPEN and esiFLEX). FLUC esiRNA (EHUFLUC) targeting firefly luciferase was used as a negative control, and KIF11 esiRNA (EHU019931) was used as a positive control for transfection and viability. siRNAs were transfected at 10 ng per well in 384-well plates (used for imaging and drug incubation) and 40 ng per well in 96-well plates (RNA extraction, quantitative real-time PCR (qRT–PCR)) with Lipofectamine RNAiMAX (Invitrogen, 13778075). For 384-well plates, both siRNAs and Lipofectamine were dispensed using a Labcyte Echo liquid handler in a randomized plate layout to control for plate effects when possible. For data presented in Figs. 3d and 5i and Extended Data Figs. 4d and 7j, LN-229 cells were incubated at 37 °C, 5% CO_2_ for 48 h after siRNA transfection before fixation, IF and RNA extraction. For Fig. 5k, after 48 h of siRNA transfection, LN-229 cells were incubated for an additional 24 h with either DMSO control or vortioxetine (10 µM) before fixing and subsequent analysis.

siRNA knockdown efficiency and relative abundance for the genes *BTG1*, *BTG2*, *JUN* and *MKI67* were measured by TaqMan Array 96-well plates (Applied Biosystems) using TaqMan Fast Advanced Master Mix (Thermo Fisher Scientific, A44360) on a QuantStudio 3 Real-Time PCR System (Applied Biosystems, A28567). Total RNA from LN-229 cells was extracted using the Direct-zol RNA MicroPrep Kit (Zymo Research, R2062) and measured using a Qubit 4 fluorometer (Thermo Fisher Scientific). cDNA was synthesized with an iScript cDNA Synthesis Kit (Bio-Rad, 1708890). For each TaqMan biological replicate assay (*n* = 3 replicates), 25 ng of cDNA per sample was used. To calculate the relative abundance of each target gene, the geometric mean Ct value of four endogenous control genes (*18S rRNA*, *GAPDH*, *HPRT* and *GUSB*) was subtracted from each [sample-target gene] Ct value to derive the deltaCt (dCt) value.

### COSTAR

COSTAR is an interpretable molecular machine learning approach that uses logistic LASSO regression in a cross-validation setting to learn a multi-linear model that identifies the minimal set of drug–target connections that maximally discriminates PCY-hit drugs from PCY-negative drugs.

Drug–target connections were retrieved from the DTC^41^. DTC is a crowd-sourced platform that integrates drug-target bioactivities curated from both literature and public databases, such as PubChem and ChEMBL. Drug–target annotations (DTC bioactivities) listed as of August 2020 were included, with the target organism limited to *Homo sapiens*. Among PCY-tested drugs in our NAD and ONCD libraries, 127 out of 132 drugs had DTC ‘bioactivity’ annotations. PTGs with biochemical associations to a given drug correspond to bioactivities with the inhibitory constant ‘KI’ as the ‘End Point Standard Type’. ePTGs include all annotated drug bioactivities. STGs downstream of ePTGs were retrieved by high-confidence protein–protein interactions annotated in the STRING database (interaction score ≥ 0.6). The final drug–target connectivity map that was used for COSTAR consisted of 127 PCY-tested drugs, 975 extended primary targets, 10,573 secondary targets and 114,517 network edges. The 127 drugs were labeled either as PCY-hits (*n* = 30, equally split across NADs and ONCDs) or as PCY-negative drugs (*n* = 97) based on the ranked mean PCY score across patients.

A 20-fold cross-validated LASSO generalized linear model was trained in MATLAB with the drug–target connectivity map as the predictor variable and PCY-hit status (hit versus negative) as the binomially distributed response variable to identify the optimal regularization coefficient (lambda) across a geometric sequence of 60 possible values. Final model coefficients were fitted using the lamba value corresponding to the minimum deviance in a cross-validation setting (Extended Data Fig. 5a). COSTAR performance was first evaluated on the training dataset, represented as a confusion matrix in Fig. 4d. Using this trained linear model, COSTAR was next used as an in silico drug screening tool to predict the PCY-hit probability (COSTAR score) based on the connectivity of 1,120,823 compounds annotated in DTC (Supplementary Data 2). For interpretability, COSTAR subscores, defined as the individual connectivity to target genes multiplied by their respective coefficients (betas) in the linear model, can be investigated in Fig. 4h and Extended Data Fig. 5c. COSTAR predictions from this in silico screen were further experimentally validated ex vivo by PCY in glioblastoma patient samples (*n* = 4) on a set of untested drugs predicted as either COSTAR-HIT (*n* = 23) or COSTAR-NEG (*n* = 25).

### DRUG-seq

High-throughput multiplexed RNA-seq was performed with the DRUG-seq method as described in Ye et al.^43^ with a few modifications. Oligonucleotides used for DRUG-seq are listed in Supplementary Table 5. Modifications to the published method are the following: (1) extraction of RNA before cDNA reverse transcription with the Zymo Direct-zol-96 RNA isolation kit (Zymo Research, R0256); (2) change of reverse transcription primers for compatibility with standard Illumina sequencing primers; (3) cDNA clean-up before library amplification performed with the DNA Clean & Concentrator-5 kit (Zymo Research, D4013); and (4) tagmentation performed with 2-ng input and sequencing library generated using the Nextera XT library prep kit (Illumina, FC-131-1024). In short, 1 × 10^4^ LN-229 cells were plated in CellCarrier 96 Ultra microplates (PerkinElmer, 6055302) and incubated overnight in reduced serum media at 37 °C, 5% CO_2_ before drug treatment. A total of 20 drugs (Supplementary Table 2) were profiled across two different timepoints (6 h and 22 h; *n* = 4 replicates per drug/timepoint). These drugs included PCY-hit NADs spanning diverse drug classes (*n* = 11), PCY-hit ONCDs (*n* = 7), PCY-negative NADs (*n* = 2) and DMSO. Cells in drug-treated 96-well plates were lysed with TRIzol reagent (Thermo Fisher Scientific, 15596018), and then subsequent cDNA and library prep was performed as described above. Finally, 100-bp (80:20) paired-end reads were generated using Illumina’s NextSeq 2000 platform.

### Calcium assays on the FLIPR platform

For FLIPR calcium assays, LN-229 or P050.C cells were seeded on poly-d-lysine-coated ViewPlate-96 microplates (PerkinElmer, 6005182) in 100 µl of medium (LN-229: 70,000 cells per well; P050.C: 20,000 cells per well) 24 h before the experiment. Fluorescent Ca^2+^ signal was measured using the Calcium 6 assay kit (Molecular Devices, 5024048) by the FLIPR Tetra (Molecular Devices) using a 470–495-nm LED excitation module and a 515–575-nm emission filter. Calcium 6 dye stock solution was prepared in 10 ml of sterile-filtered nominal Ca^2+^ free (NCF) modified Krebs buffer containing 117 mM NaCl, 4.8 mM KCl, 1 mM MgCl_2_, 5 mM D-glucose and 10 mM HEPES (pH 7.4) stored as 500-µl aliquots at −20 °C. Before each experiment, the dye stock was freshly diluted 1:10 in NCF Krebs buffer, and, after removing the medium from the cells, 50 µl of the diluted dye was applied per well followed by incubation at 37 °C for 2 h in the dark. For the assay setup outlined in Fig. 5e, cells were treated with their respective PCY-drug after a period of equilibration in 2 mM calcium-containing buffer. For fold change calculations presented in Fig. 5f and Extended Data Fig. 7b, normalized calcium levels for each drug were calculated by averaging calcium levels after drug treatment (400–600-s interval) divided by the basal level of calcium before drug administration (200–300-s interval). In the ER Ca^2+^ store release assay, stable baselines were established for 50 s before 50 µl of 2 µM (2×) thapsigargin (Sigma-Aldrich, T9033) or 40 µM (2×) drug solutions freshly prepared in NCF Krebs buffer were robotically dispensed. Next, the cells were incubated, and fluorescence was monitored in the presence of thapsigargin or drugs for another 5 min. In the extracellular Ca^2+^ uptake assay, after initial recording of the baseline, 50 µl of 4 mM CaCl_2_ (2×) prepared in NCF Krebs buffer was dispensed onto the cells to re-establish a physiological 2 mM calcium concentration, and the fluorescence was monitored for 5 min. Next, 60 µM (3×) drug solutions freshly prepared in Krebs buffer containing 2 mM CaCl_2_ were robotically dispensed, and fluorescence was recorded for an additional 4 min. The raw data were extracted with ScreenWorks software version 3.2.0.14. The values represent average fluorescence level of the Calcium 6 dye measured over arbitrary selected and fixed timeframes.

### Calcium imaging using the Fura-2 calcium indicator

Glioblastoma cell lines (LN-229 and LN-308) and PDCs (P024.C, P040.C, P049.C and P050.C lines) were seeded in six-channel µ-Slide VI 0.4 ibiTreat (ibidi, 80606), with 30,000–100,000 cells per channel and up to three channels per slide. Seeded cells were cultured in these chamber slides 1–2 d before the experiment to achieve approximately 70–80% confluency. Before dye loading of the Fura-2 AM calcium indicator (Thermo Fisher Scientific, F1221), cells were washed two times with HEPES-buffered Krebs-Ringer Solution (referred to as Krebs buffer; Thermo Fisher Scientific, 67795.K2). Cell permeant Fura-2 dye resuspended in DMSO was incubated with cells (1 μM solution in Krebs buffer) for 15 min at 37 °C, 5% CO_2_ in a dark humidified incubator and washed three times with Krebs buffer before imaging. All subsequent calcium imaging and drug perfusion were performed in Krebs buffer.

Live-cell calcium imaging was performed at ×20 magnification (S Fluor ×20 NA 0.75 objective) on a Nikon Ti2-E inverted microscope equipped with a Nikon DS-FI3 color camera (2,880 × 2,048 pixels, 2.4 μm × 2.4 μm), color BF camera, motorized fast emission filter wheels (Sutter Instrument) and a FURA dichroic mirror. FURA filterset specifications include: LED 1 (excitation window 1), 340/26; LED 2 (excitation window 2), 387/11; and an emission filter, 510/84. 2 × 2 binned images were acquired every 2 s throughout an imaging time of 10 min per experiment. CO_2_ levels and temperature were controlled by an Okolab box type incubation system. Vortioxetine (20 μM solution) was manually administered on the chamber slide. Timepoint of drug addition was, on average, between 125 s and 140 s after the start of imaging. Downstream image analysis was performed with ImageJ and R. In ImageJ, circular regions of interest (ROIs) were manually selected for each cell present in the first image frame of each experiment’s time series as well as five background ROIs to calculate the mean background intensity. For both the 340-nm and 380-nm channels, mean pixel intensities across each cell ROI and image frame were measured. Subsequently in R, mean background intensity was subtracted from each cell ROI before further downstream analysis. Cell ROIs with more than five image timeframes exhibiting a signal lower than background (lower one percentile of Fura-2 intensities across cells in the first 30 s of imaging) were excluded from the analysis. Timepoint of vortioxetine addition was determined either by outlier detection or by manual inspection between 120 s and 150 s after the start of imaging, and this single timeframe was assigned to N/A (not applicable) to exclude the possibility of imaging artifacts impeding the analysis. The raw Fura-2 calcium signal was defined as the ratio of 340/380 intensity. The mean change in calcium signal after vortioxtine treatment was defined as the baseline signal before drug treatment subtracted from the calcium signal after vortioxetine treatment, each averaged across a 120-s time window. Normalized Fura-2 calcium signal corresponds to the baseline signal subtracted from the raw signal on a cell ROI basis. The presence (Ψ) or absence (Ø) of oscillatory calcium signaling was determined by peak detection analysis. If a cell ROI had more than one or two peaks detected within its respective time span (baseline versus after vortioxetine drug treatment), the response type was assigned as oscillatory.

### Electrophysiology

LN-229 and LN-308 glioblastoma cell lines were seeded at approximately 40% confluence in 35-mm Petri dishes (CLS430165, Corning). Whole-cell patch-clamp recordings were performed with a HEKA EPC10 USB amplifier using the following solutions: extracellular (in mM): 140 NaCl, 2 MgCl_2_, 2 CaCl_2_, 10 HEPES, 3 KCl, 10 D-glucose, pH 7.4; pipette (in mM): 4 NaCl, 120 K-gluconate, 10 HEPES, 10 EGTA, 3 Mg-ATP, 0.5 CaCl_2_, 1 MgCl_2_, pH 7.2 (liquid junction correction 12 mV). Patch pipettes (~10 MOhm) were pulled from borosilicate glass capillaries (Harvard Apparatus, 30-0038) using a two-step vertical pipette puller PC 100 (Narishige) and further fire-polished using a homemade microforge. Membrane voltage was measured during 10 s (current-clamp mode), and currents elicited upon changes in voltage (voltage-clamp mode) were assessed by keeping cells at −50 mV for 300 ms, followed by stepwise increments of +20 mV during 1,000 ms (−120 mV to +100 mV) and ending with −50 mV for 300 ms. Current-clamp and voltage-clamp protocols were executed automatically every minute during the experiment. Cells were kept at their respective membrane voltage (voltage clamp) in between protocols. For every cell, a 5-min control period was recorded after achieving whole cell followed by a 10-min recording with vortioxetine, 10 μM treatment. Average steady-state current and membrane voltage were calculated during 80% of recorded time. A linear mixed-effects model was fitted by: ‘Current density ~ Command voltage (Vcmd)* Condition (Cond.) + (1|Cell ID)’ to assess how command voltage and condition influence current density. Summary statistics are reported in Extended Data Fig. 7i.

### Incucyte live-cell imaging

In total, 2.5 × 10^3^ LN-229 cells per well were plated in CellCarrier 96 Ultra microplates (PerkinElmer, 6055302) 24 h before the experiment and transfected with *BTG1*, *BTG2* and *FLUC* (−) MISSION esiRNAs (Sigma-Aldrich, Euphoria Biotech, 40 ng per well) using Lipofectamine RNAiMAX (Invitrogen, 13778075). Real-time confluence of cell cultures (*n* = 4 replicate wells per condition) was monitored by imaging every 2 h for 7 d at ×10 magnification with the ‘phase’ channel using the Incucyte live-cell analysis system S3 (Sartorius). Automatic image segmentation and analysis of the phase-contrast images was performed by the Incucyte base analysis software (version 2020B).

### Timecourse RNA-seq library preparation and sequencing

LN-229 cells were seeded at 2 × 10^5^ cells per well in six-well Nunc Cell-Culture Treated Multidishes (Thermo Fisher Scientific, 140675) and incubated overnight in reduced serum media at 37 °C, 5% CO_2_ before drug treatment. The following day, vortioxetine (AvaChem Scientific, 3380) was manually added to each well at a final concentration of 20 µM. At the start of the experiment, LN-229 cells that were not treated with vortioxetine were collected as the 0-h timepoint. After 3, 6, 9, 12 and 24 h following vortioxetine treatment, drug-containing media were removed, and cells were collected in TRIzol reagent (Thermo Fisher Scientific, 15596018). Total RNA was isolated using Direct-zol RNA MicroPrep Kit (Zymo Research, R2062), and RNA quality and quantity were determined with an Agilent 4200 TapeStation. Sample RNA integrity number (RIN) scores ranged from 5.9 to 10 (mean RIN, 9.33). RNA input was normalized to 300–400 ng, and RNA libraries were prepared using the Illumina TruSeq stranded mRNA library prep. Then, 100-bp single-end reads were generated using Illumina’s NovaSeq 6000 platform with an average sequencing depth of approximately 50 million reads per replicate. Reads were mapped and aligned to the reference human genome assembly (GRCh38.p13) using STAR/2.7.8a, and counts were extracted using ‘featureCounts’. Subsequent read normalization (variance stabilizing transformation, vsd-normalized counts) and RNA-seq analysis, including differential expression analysis, was performed with the R package ‘DESeq2’^72^.

### Timecourse proteomics and phosphoproteomics

Cell preparation and vortioxetine treatment were performed as in the ‘Timecourse RNA-seq library preparation and sequencing’ subsection except that cell numbers were scaled to be seeded in T-150 culture flasks, and three timepoints were measured (0 h, 3 h and 9 h). Peptides were prepared using the PreOmics iST kit on the PreON (HSE AG) programmed to process eight samples in parallel. Cell pellets were resuspended in 50 µl of lysis buffer and denatured for 10 min at 95 °C, followed by 3 h of digestion with trypsin and Lys-C. Peptides were dried in a speed-vac (Thermo Fisher Scientific) for 1 h before being resuspended in LC-LOAD buffer at a concentration of 1 μg μl^−1^ with iRT peptides (Biognosys).

Samples were analyzed on an Orbitrap Lumos mass spectrometer equipped with an Easy-nLC 1200 (both Thermo Fisher Scientific). Peptides were separated on an in-house packed 30-cm RP-HPLC column (Michrom Bioresources, 75 μm i.d. × 30 cm; Magic C18 AQ 1.9 μm, 200 Å). Mobile phase A consisted of HPLC-grade water with 0.1% formic acid (FA); mobile phase B consisted of HPLC-grade acetonitrile (ACN) (80%) with HPLC-grade water and 0.1% (v/v) FA. Peptides were eluted at a flow rate of 250 nl min^−1^ using a nonlinear gradient from 4% to 47% mobile phase B in 228 min. For data-independent acquisition (DIA), DIA-overlapping windows were used, and a mass range of *m*/*z* 396–1,005 was covered. The DIA isolation window size was set to 8 *m*/*z* and 4 *m*/*z*, respectively, and a total of 75 or 152 DIA scan windows were recorded at a resolution of 30,000 with an AGC target value set to 1,200%. Higher-energy collisional dissociation (HCD) fragmentation was set to 30% normalized collision. Full mass spectra were recorded at a resolution of 60,000 with an AGC target set to standard and the maximum injection time set to auto. DIA data were analyzed using Spectronaut version 14 (Biognosys). MS1 values were used for quantification, and peptide quantity was set to mean. Data were filtered using *q* value sparse with a precursor and a protein *q* value cutoff of 0.01 FDR. Interference correction and local cross-run normalization was performed. For PRM measurements, peptides were separated by reverse-phase chromatography on a 50-cm ES803 C18 column (Thermo Fisher Scientific) that was connected to a Easy-nLC 1200 (Thermo Fisher Scientific). Peptides were eluted at a constant flow rate of 200 nl min^−1^ with a 117-min nonlinear gradient from 4% to 52% buffer B (80% ACN, 0.1% FA) and 25–50% B. Mass spectra were acquired in PRM mode on an Q Exactive HF-X Hybrid Quadrupole-Orbitrap MS system (Thermo Fisher Scientific). The MS1 mass range was 340–1,400 *m*/*z* at a resolution of 120,000. Spectra were acquired at 60,000 resolution (automatic gain control target value 2.0 × 10^5^). Normalized HCD collision energy was set to 28% and maximum injection time to 118 ms. Monitored peptides were analyzed in Skyline version 20, and results were uploaded to PanoramaWeb.

For phosphopeptide enrichment, protein lysate from LN-229 cells was prepared using a deoxycholate-based buffer. Five hundred micrograms of vortioxetine-treated cells at each timepoint (*n* = 3 replicates) were used as starting material. A tryptic digest was performed for 16 h. Samples were then purified on MACROSpin C18 columns (Harvard Apparatus). Phosphopeptides were specifically enriched using IMAC cartridges on the Bravo AssayMAP liquid handling platform (Agilent). Samples were dissolved in 160 μl of loading buffer (80% ACN, 0.1% trifluoroacetic acid (TFA)). Then, the AssayMAP phosphoenrichment protocol was performed with slight modifications. After purification, dried peptides were resuspended in LC buffer and subjected to DDA-MS on a Q Exactive H-FX mass spectrometer equipped with an Easy-nLC 1200 (both Thermo Fisher Scientific). Peptides were separated on an ES903 column (Thermo Fisher Scientific, 75 μm i.d. × 50 cm; particle size 2 μm). Mobile phase A consisted of HPLC-grade water with 0.1% FA; mobile phase B consisted of HPLC-grade ACN (80%) with HPLC-grade water and 0.1% (v/v) FA. Peptides were eluted at a flow rate of 250 nl min^−1^ using a nonlinear gradient from 3% to 56% mobile phase B in 115 min. MS1 spectra were acquired at a resolution of 60,000 with an AGC target value of 3^6^ and a maximum injection time of 56 ms. The scan range was between 350 *m*/*z* and 1,650 *m*/*z*. A data-dependent top 12 method was used with a precursor isolation window of 1.3 *m*/*z*. MS/MS scans were acquired with normalized collision energy of 27 at a resolution of 15,000. AGC target was 1^5^ with a maximum injection time of 22 ms. Dynamic exclusion was set to 30 s. Data analysis was performed using FragPipe (version 19.1) with the LFQ-phospho workflow^73^. Min site localization probability was set to 0.75 in IonQuant^74^. Statistical analysis was performed on the phosphoprotein-filtered combined protein output in FragPipe-Analyst.

### Clonogenic survival assay

Adherent cells (LN-229: 50 cells; LN-308: 300 cells) were seeded in 96-well plates (*n* = 6 wells per condition; 100 µl of medium) and incubated overnight. On the following day, medium was replaced by fresh medium containing indicated final concentrations of vortioxetine or DMSO. Glioblastoma-initiating cells (ZH-161 and ZH-562; 500 cells) were seeded in 75 µl of medium and incubated overnight. Treatment was initiated by addition of 75 µl of medium containing 2× concentrated vortioxetine or DMSO to reach indicated final concentrations. DMSO concentration was kept at 0.5% for all treatments and controls. After treatment addition, cells were cultured for 11 d (LN-229) to 13 d (other cell lines), and clonogenic survival was estimated from a resazurin-based assay^75^ using a Tecan M200 PRO plate reader (λEx = 560 nm / λEm = 590 nm).

### Collagen-based spheroid invasion assay

Spheroid invasion assay was performed as described (Kumar et al.^76^). In brief, 2,000 cells were seeded cell-repellent 96-well U-bottom plates (Greiner Bio-One, 650979, *n* = 6 wells per condition) and incubated for 48 h to allow spheroid formation. Subsequently, 70 µl of medium was removed, and spheroids were overlaid with 70 µl of 2.5% Collagenase IV (Advanced Biomatrix, 5005-B) in 1× DMEM containing sodium bicarbonate (Sigma-Aldrich, S8761), and collagen was solidified in the incubator for 2 h. Collagen-embedded spheroids were then overlaid with 100 µl of chemoattractant (NIH-3T3-conditioned medium) containing 2× concentrated vortioxetine/DMSO (0.5% final DMSO concentration across conditions) and incubated for 36 h. Spheroids were stained with Hoechst, and images were acquired on a MuviCyte imaging system (PerkinElmer, HH40000000) using a ×4 objective. Images were contrast enhanced and converted to binary using ImageJ/Fiji and quantified with automated Spheroid Dissemination/Invasion counter software (aSDIcs), which quantifies the migration distance from the center of the spheroid for each detected cell nucleus^76^.

### In vivo drug testing

All animal experiments were performed under the guidelines of Swiss federal law on animal protection and were approved by the cantonal veterinary office (ZH98/2018). CD1 female nu/nu mice (Janvier) of 6–12 weeks of age were used in all experiments, and 100,000 LN-229-derived or 150,000 ZH-161-derived cells were implanted^77^. Mice were euthanized when they exhibited neurological symptoms or a mouse grimace scale score of 2 (ref. ^78^). We confirm that these criteria were not exceeded. Mice were housed in groups of five mice per cage in the animal facility of LASC Zurich and kept in transparent plastic Eurostandard Type III cages measuring 425 × 266 × 155 mm. The cages contained autoclaved, dust-free sawdust bedding (80–90 g per cage) and one Nestlet (5 × 5 cm). The mice were fed a pelleted and extruded Kliba No. 3436 mouse diet (Provimi Kliba) ad libitum and had unrestricted access to sterilized drinking water. The room maintained a 12-h light/dark cycle with artificial light. The temperature was 21 ± 1 °C, and the relative humidity was 50 ± 5%.

Test-naive mice were randomly assigned to drug treatment groups for experiments (in vivo drug treatment Trials I–V). Tumor-bearing mice were treated from day 5 to day 21 after tumor implantation with intraperitoneally administered vortioxetine daily 10 mg kg^−1^, citalopram daily 10 mg kg^−1^, paliperidone daily 5 mg kg^−1^, apomorphine daily 5 mg kg^−1^, aprepitant daily 20 mg kg^−1^, brexpiprazole daily 1 mg kg^−1^, chlorpromazine three times per week 10 mg kg^−1^, TMZ 50 mg kg^−1^ for five consecutive days, CCNU 20 mg kg^−1^ at day 7 and day 14 after tumor implantation or daily DMSO control. MRI was performed with a 4.7T imager (Bruker BioSpin) when the first mouse became symptomatic for in vivo Trials I–III or a 7T imager (Bruker BioSpin) at days 12, 25, 38 and 48 after tumor implantation for in vivo Trial IV. Coronal T2-weighted images were acquired using ParaVision 360 (Bruker BioSpin). Tumor regions were identified manually by two independent raters, and maximum perimeter was outlined and quantified using MIPAV (11.0.7).

For immunohistochemistry analysis, mouse brains were embedded in Shandon Cryochrome (Thermo Fisher Scientific) and were cut horizontally by 8-μm steps until reaching the tumor. Tissue sections were stained for 1 s with 0.4% methylene blue and rinsed with deionized water (2 × 10 dips) to confirm tumors (when present) under the microscope. Sections were stored in dark dry boxes overnight before being stored at −80 °C. Sections were fixed with 4% PFA (Sigma-Aldrich, F8775) in PBS, blocked in 5% FBS and 0.1% Triton containing PBS and stained overnight at 4 °C in blocking solution with DAPI and the following antibodies and dilutions: Alexa Fluor 488 anti-vimentin (1:500, BioLegend, 677809, clone O91D3), anti-Ki67 (1:300, Cell Signaling Technology, 9129S, clone D3B5) and goat anti-rabbit IgG (H + L) highly cross-adsorbed secondary antibody, Alexa Fluor Plus 647 (1:500, Thermo Fisher Scientific, A32733). Imaging was performed by ×20 fluorescence imaging using the Pannoramic 250 slide scanner (3DHISTECH).

### Statistical analysis

For prospectively sampled patient material, no sample size determination was performed a priori as the effect size and variability of ex vivo drug response among patients were unknown before the study. Our sample sizes are similar to other published glioblastoma studies investigating the heterogeneity of patient samples and/or patient-derived explants^4,38,60^. For all other statistical analysis, their respective tests and significance values are reported in each corresponding figure panel and/or Methods. For linear correlations, Pearson correlation coefficients with two-tailed *P* values are annotated. When the Student’s *t*-test was used for comparisons between groups (for example, drug treatment versus control), data distribution was assumed to be normal, but this was not formally tested. The Wilcoxon test was also used as a non-parametric equivalent. For matched patient samples or cells, paired *t*-tests or Wilcoxon tests were used. Unless otherwise stated, multiple testing correction was performed using either the Holm method for comparisons of fewer than 20 data points or the FDR procedure for larger datasets.

### Reporting summary

Further information on research design is available in the Nature Portfolio Reporting Summary linked to this article.

## Online content

Any methods, additional references, Nature Portfolio reporting summaries, source data, extended data, supplementary information, acknowledgements, peer review information; details of author contributions and competing interests; and statements of data and code availability are available at 10.1038/s41591-024-03224-y.

## Supplementary information

Supplementary Information Supplementary Figs. 1 and 2.

Reporting Summary

Supplementary Tables.

Supplementary Data 1 Table summarizing results from Oncomine Comprehensive Assay version 3 performed for the prospective glioblastoma cohort (*n* = 27 patients). The Oncomine assay is a targeted NGS method enabling the detection of SNVs, CNVs, gene fusions and indels commonly present in cancers (*n* = 161 genes). (Sheet 1) ‘Result_Summary’ provides an overview of genetic point mutations, indels, CNVs and gene fusions detected in the cohort, with the color legend corresponding to the respective category of genetic alterations. (Sheet 2) ‘Mutations’ summarizes pathogenic or likely pathogenic mutations and mutations of unknown significance based on previous literature and the ClinVar database^72^. (Sheet 3) ‘CNVs’ summarizes CNVs detected in the cohort, with selection criteria of high or low confidence gain/loss calls provided with the table. (Sheet 3) ‘Gene_Fusions’ summarizes fusion genes detected in the cohort. Study IDs found in this table can be decoded in Supplementary Table 1.

Supplementary Data 2 Table of in silico COSTAR drug screening results for 1,120,823 chemical compounds annotated in DTC. The file is provided as a CSV file format, compressed as a ZIP file to reduce storage. The table includes four columns: the CHEMBL ID for each compound, the compound name as annotated in DTC (‘COMPOUND NAME’), the COSTAR score (‘COSTAR_SCORE’) and the drug type (‘DRUG_TYPE’). The drug type annotation indicates which drugs were used in COSTAR training as PCY-negative of the NAD library (‘PCY_NEG_NAD’), as PCY-negative of the ONCD library (‘PCY_NEG_ONCD’), as PCY-hit of the NAD library (‘PCY_HIT_NAD’), as PCY-hit of the ONCD library (‘PCY_HIT_ONCD’), as predicted COSTAR-HIT selected for experimental validation (‘COSTAR_HIT’) and as predicted COSTAR-NEG selected for experimental validation (‘COSTAR_NEG’).

Supplementary Video 1 Ratiometic Fura-2 calcium indicator-based live-cell calcium imaging at baseline (BASELINE) and after vortioxetine treatment (+VORT). Representative videos of two PDC lines: PDC P024.C (left) and P049.C (right). Images are pseudocolored according to the 340/380-nm Fura-2 intensity ratio, and scaling is adjusted for visualization purposes. Images are taken at 2-s intervals.

Supplementary Video 2 Confluency of LN-229 cells measured by IncuCyte live-cell imaging (every 2 h) across 7 d in two siRNA knockdown conditions (*BTG1* and *BTG2*) and a negative firefly luciferase control (*FLUC*). BF segmentation mask is in yellow.

## Source data

Source Data Fig. 1 Statistical source data.

Source Data Fig. 2 Statistical source data.

Source Data Fig. 3 Statistical source data.

Source Data Fig. 4 Statistical source data.

Source Data Fig. 5 Statistical source data.

Source Data Fig. 6 Statistical source data.

Source Data Extended Data Fig. 1 Statistical source data.

Source Data Extended Data Fig. 2 Statistical source data.

Source Data Extended Data Fig. 3 Statistical source data.

Source Data Extended Data Fig. 4 Statistical source data.

Source Data Extended Data Fig. 5 Statistical source data.

Source Data Extended Data Fig. 6 Statistical source data.

Source Data Extended Data Fig. 7 Statistical source data.

Source Data Extended Data Fig. 8 Statistical source data.

Source Data Extended Data Fig. 9 Statistical source data.

Source Data Extended Data Fig. 10 Statistical source data.

## Data Availability

All transcriptomics data generated in this study, including scRNA-seq, bulk RNA-seq and DRUG-seq datasets, have been deposited in the National Center for Biotechnology Informationʼs Gene Expression Omnibus (GEO; https://www.ncbi.nlm.nih.gov/geo/) under the following accession numbers: GSE214965 (DRUG-seq; multiplexed RNA-seq of 20 drugs, two timepoints); GSE214966 (scRNA-seq; four patients at baseline); GSE214967 (scRNA-seq; patient sample after vortioxetine versus DMSO treatment); and GSE214968 (RNA-seq; vortioxetine timecourse). Previously published scRNA-seq datasets analyzed in this study are publicly available at the GEO under the following accession numbers: GSE117891 and GSE131928. The publicly available GRCh38 human reference genome was used to align RNA-seq reads. Proteomics and phosphoproteomics data can be accessed via Panorama (https://panoramaweb.org/GlioB.url). DIA and phosphopeptide enrichment datasets are available from MASSIVE (ftp://massive.ucsd.edu/v04/MSV000090357/). Drug–target annotations and protein–protein interaction data were retrieved from the following publicly available databases: Drug Target Commons (DTC; https://drugtargetcommons.fimm.fi/) and STRING (https://string-db.org/). Other publicly available databases used in this study include DAVID (https://david.ncifcrf.gov/), KEGG (https://www.genome.jp/kegg/), Gene Ontology (http://geneontology.org/) and PathwayNet (http://pathwaynet.princeton.edu/). Data provided in supplementary tables include ex vivo drug response of glioblastoma cells (pharmacoscopy scores; Supplementary Table 2), transcriptome-wide neural specificity and patient specificity scores derived from three scRNA-seq datasets (Supplementary Table 3) and in silico COSTAR drug screening results across 1,120,823 compounds (Supplementary Data 2). Source data are provided with this paper.

## Code availability

Code for de-multiplexing of DRUG-seq data can be found on GitHub at https://github.com/RebekkaWegmann/drugseq_toolbox. COSTAR code and example data are available at https://www.snijderlab.org/resources/COSTAR/. Image analysis was performed using the open-source CellProfiler package available at https://www.cellprofiler.org. All other analyses were performed using standard MATLAB R2019a–R2023a and R 3.6.0–4.3.0 code.
