# Supplementary material for: High-throughput identification of repurposable neuroactive drugs with potent anti-glioblastoma activity
Source: Nat Med. 2024 Sep 20;30(11):3196–208. doi: 10.1038/s41591-024-03224-y (PMC11564103; doi:10.1038/s41591-024-03224-y)
Supplement: Supplementary file 1 — Supplementary Figs. 1 and 2. [file 41591_2024_3224_MOESM1_ESM.pdf]

nature medicine

Supplementary information

https://doi.org/10.1038/s41591-024-03224-y

High-throughput identification of repurposable neuroactive drugs with potent anti-glioblastoma activity

In the format provided by the authors and unedited

Supplementary Figure 1

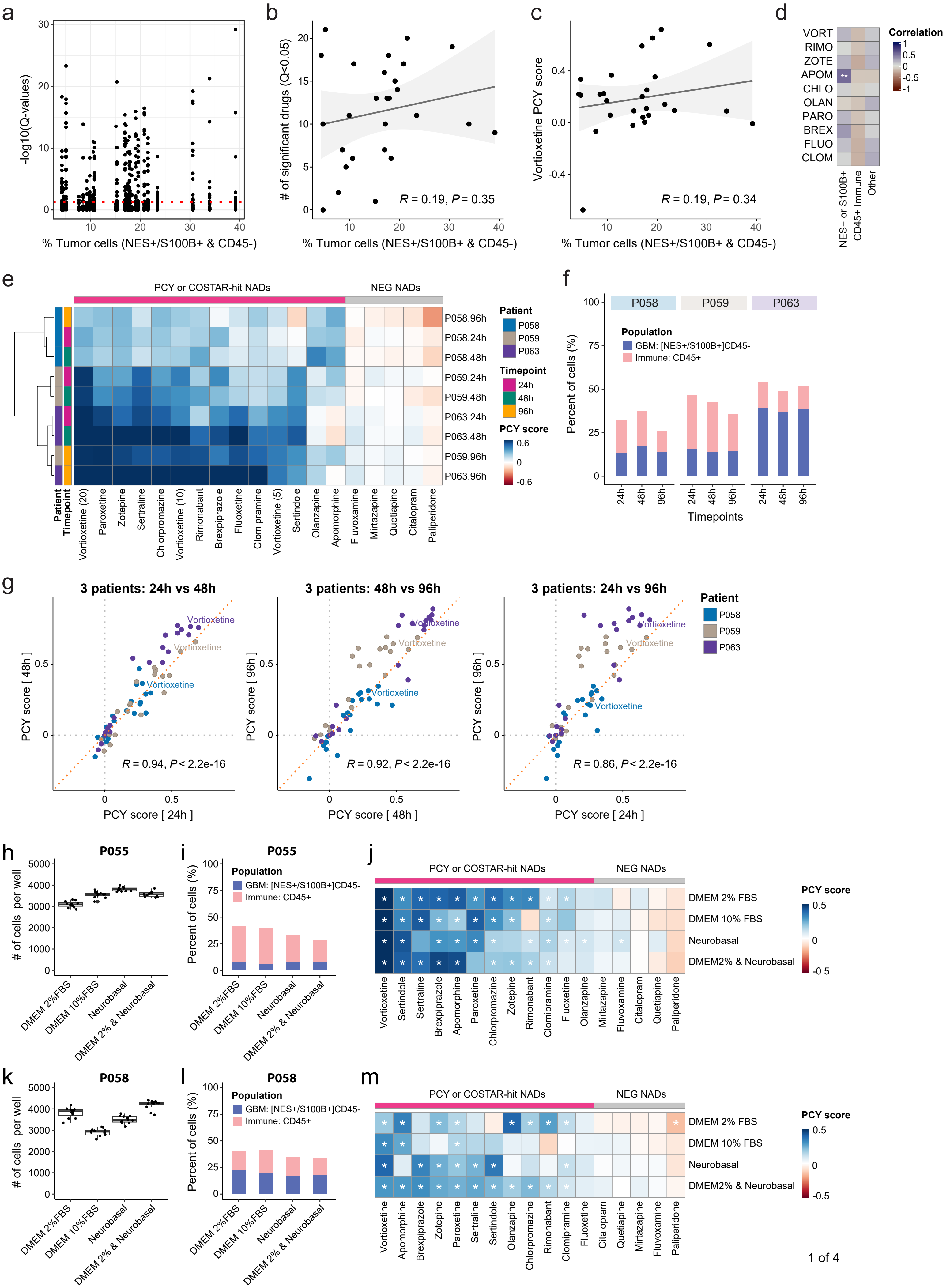

Supplementary Figure 1: Analysis of the possible effect of tumor content, assay time point, and culture conditions on the pharmacoscopy readout

a, Analysis of the statistical power (y-axis; -log10(FDR-adjusted Q-value)) of drug responses per sample reflecting effect size and technical variance between replicate wells, and **b,** the number of significant 'on-target' drugs per sample (y-axis) as a function of each patient sample's tumor content (x-axis for both plots; glioblastoma population defined by [Nestin+ or S100B+] and CD45-). This analysis demonstrates that neither statistical power nor the number of significant hits is confounded by the tumor content. **c,** Lack of association of Vortioxetine *ex vivo* response (y-axis) with tumor content (x-axis). **b, c,** Linear regression line with a 95% confidence interval. Pearson correlation coefficients with two-tailed *P*-values annotated. **d,** Expansion of the analysis in **c,** now extended to top neuroactive drug hits (rows). Pearson correlations of marker-based sample composition at baseline (columns) with the (S100B+/Nestin+ and CD45-) PCY scores for each respective drug across patients (n=27 patients). **e-g,** Analysis of neuroactive drug responses across 3 different drug incubation time points (24, 48, and 96 hours following surgery) for a subset of top- and bottom-ranking NADs (n=17 drugs) across 3 glioblastoma patient samples (P058, P059, P063). **e,** Correlation-based clustered heatmap of overall drug response revealed a high degree of drug response similarity per patient sample across time points. The top mean ranking NAD across time points was Vortioxetine, followed by Paroxetine and Zotepine, while PCY-negative NADs identified at 48 hours did not show efficacy neither at the 24 nor 96-hour time point. **f,** The relative abundance of sample composition (GBM; glioblastoma cells and immune cells) did not significantly differ between the timepoints. **g,** Correlation of neuroactive drug responses across patients and between drug incubation time points reveals a high degree of similarity, with preservation of relative drug sensitivity differences between patients (e.g. relative ranking of Vortioxetine). Pearson correlation coefficients with two-tailed *P*-values annotated. **h-k,** Experimental comparison of neuroactive drug screening results for a subset of top- and bottom-ranking NADs similarly to **e,** between 4 different media conditions (DMEM 2%, DMEM 10%, Neurobasal, and a 50:50 mix of DMEM and Neurobasal) in two surgical patient samples (P055, P058) at the 48 hour time point. Media condition abbreviations are as follows: DMEM 2%, DMEM supplemented with 2% FBS and 25mM HEPES; DMEM 10%, DMEM supplemented with 10% FBS and 25mM HEPES; Neurobasal, Neurobasal supplemented with B27, bFGF, EGF, and 2 mM L-glutamine. All media conditions supplemented with 1% PenStrep. Drug screening results relating to **h-i,** patient P055 sample and **j-k,** patient P058 sample. Neither **h, j,** sample composition in DMSO control (n=12 replicate wells; right panel, mean of DMSO wells), nor **i, k,** the drug responses (n=4 wells/drug) were strongly affected by altering between these 4 different culture conditions. Two-tailed t-test compared to DMSO. * indicates FDR-adjusted *P*-value < 0.05. The ranking of drugs across conditions per patient sample identifies Vortioxetine as the consistent top neuroactive drug, and differences in the drug sensitivities between the two patient samples were maintained despite altering the culture conditions.

Supplementary Figure 2

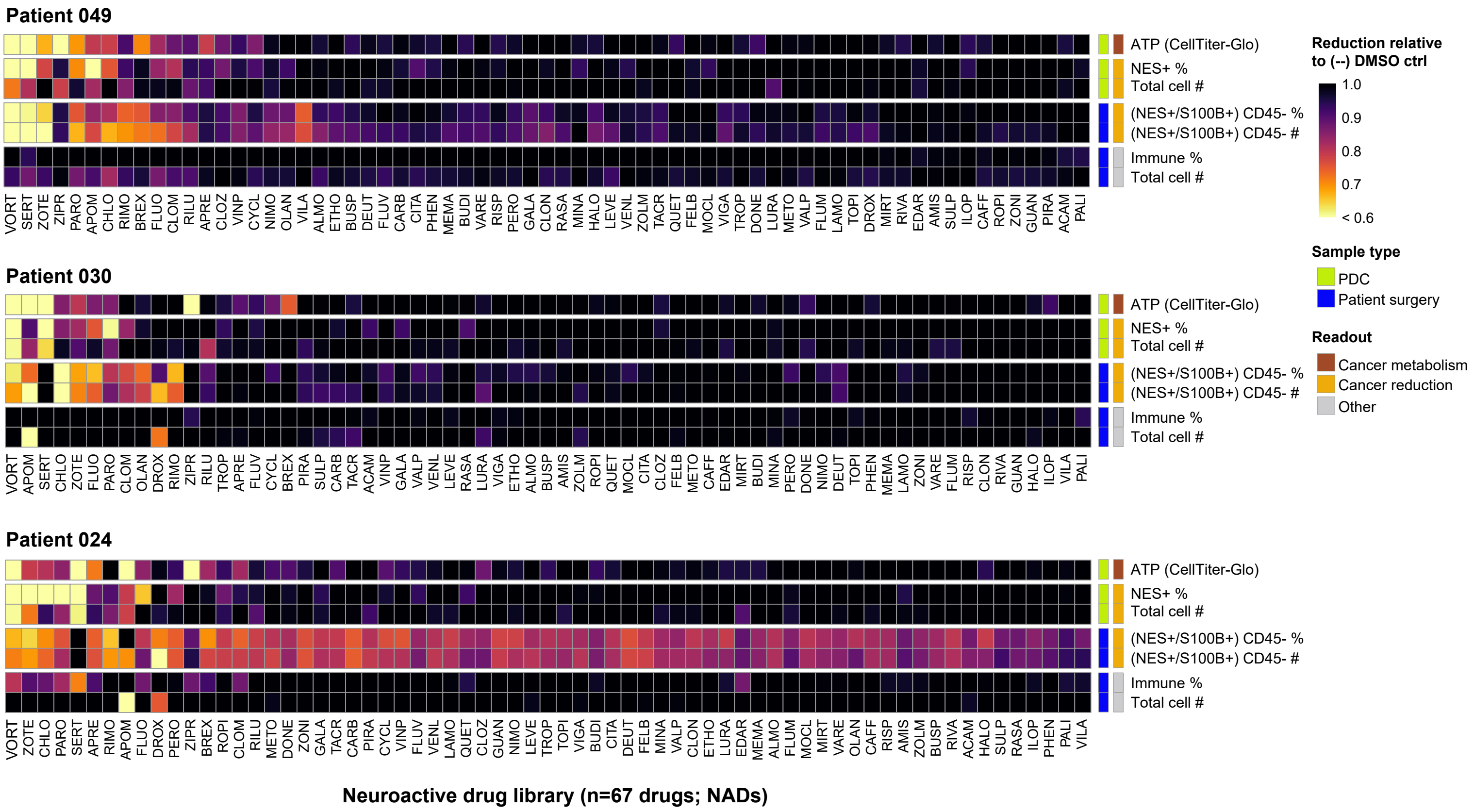

Supplementary Figure 2: Patient-matched comparison of neuroactive drug screening results between different cell culture systems and readouts including metabolic activity

Patient-matched comparison of drug screening results (n=3 patients; n=67 neuroactive drugs; NAD library) between different cell cultures and readouts. This includes, first, short term patient-derived cell cultures (PDCs) measuring: a) the metabolic activity and viability with a ATP-based luminescence assay (CellTiter-Glo), b) the relative fraction of Nestin+ cells by automated microscopy (NES+ %, and c) the total cell number by automated microscopy (Total cell #). Second, 'immediate' patient surgery material by pharmacoscopy, measuring: a) the relative fraction of cancer cells ((NES+/S100B+) CD45- %), please note that 1 - this readout is the "PCY score" used in the manuscript, b) the absolute number of cancer cells ((NES+/S100B+) CD45- #), c) the relative fraction of immune cells (Immune %), and d) the total cell number (Total cell #). The heatmap displays the results per patient and drug (columns) ordered by mean ranking across the different cancer cell viability readouts. In each of the three patients, good concordance between the five different readouts that capture cancer cell viability in response to drug treatment is observed.
