# Supplementary material for: High-throughput identification of repurposable neuroactive drugs with potent anti-glioblastoma activity
Source: Nat Med. 2024 Sep 20;30(11):3196–208. doi: 10.1038/s41591-024-03224-y (PMC11564103; doi:10.1038/s41591-024-03224-y)
Supplement: Supplementary file 2 — Reporting Summary [file 41591_2024_3224_MOESM2_ESM.pdf]

nature portfolio

Corresponding author(s): Berend Snijder

Last updated by author(s): Aug 19, 2024

Reporting Summary

Nature Portfolio wishes to improve the reproducibility of the work that we publish. This form provides structure for consistency and transparency in reporting. For further information on Nature Portfolio policies, see our Editorial Policies and the Editorial Policy Checklist.

Statistics

For all statistical analyses, confirm that the following items are present in the figure legend, table legend, main text, or Methods section.

n/a	Confirmed	
☐	☒	The exact sample size (*n*) for each experimental group/condition, given as a discrete number and unit of measurement
☐	☒	A statement on whether measurements were taken from distinct samples or whether the same sample was measured repeatedly
☐	☒	The statistical test(s) used AND whether they are one- or two-sided<br>*Only common tests should be described solely by name; describe more complex techniques in the Methods section.*
☐	☒	A description of all covariates tested
☐	☒	A description of any assumptions or corrections, such as tests of normality and adjustment for multiple comparisons
☐	☒	A full description of the statistical parameters including central tendency (e.g. means) or other basic estimates (e.g. regression coefficient) AND variation (e.g. standard deviation) or associated estimates of uncertainty (e.g. confidence intervals)
☐	☒	For null hypothesis testing, the test statistic (e.g. *F*, *t*, *r*) with confidence intervals, effect sizes, degrees of freedom and *P* value noted<br>*Give P values as exact values whenever suitable.*
☒	☐	For Bayesian analysis, information on the choice of priors and Markov chain Monte Carlo settings
☒	☐	For hierarchical and complex designs, identification of the appropriate level for tests and full reporting of outcomes
☐	☒	Estimates of effect sizes (e.g. Cohen's *d*, Pearson's *r*), indicating how they were calculated

Our web collection on statistics for biologists contains articles on many of the points above.

Software and code

Policy information about availability of computer code

Data collection	All data was collected with commercially available and/or previously published methods. See Methods section and Supplementary Information for further details.
Data analysis	MATLAB R2019a-R2020a, R Studio, R version 4.1.0, R package ‘infercnv’ (1.18.0), CellProfiler 2.2.0, ImageJ, FlowJo 10.4.2, 10x Genomics Cell Ranger versions 3.0.1 and 6.1.1, Torrent Suite Software (Ion Reporter, 5.12 and after), ScreenWorks software version 3.2.0.14, Spectronaut version 14, Incucyte base analysis software version 2020B, Spheroid Dissemination/Invasion counter software (aSDIcs), MIPAV 11.0.7.

For manuscripts utilizing custom algorithms or software that are central to the research but not yet described in published literature, software must be made available to editors and reviewers. We strongly encourage code deposition in a community repository (e.g. GitHub). See the Nature Portfolio guidelines for submitting code & software for further information.

Data

Policy information about availability of data

All manuscripts must include a data availability statement. This statement should provide the following information, where applicable:

- Accession codes, unique identifiers, or web links for publicly available datasets
- A description of any restrictions on data availability
- For clinical datasets or third party data, please ensure that the statement adheres to our policy

All transcriptomics data generated in this study including single-cell RNA-Seq, bulk RNA-Seq, and DRUG-Seq datasets have been deposited in the public repository

NCBI Gene Expression Omnibus (GEO; https://www.ncbi.nlm.nih.gov/geo/) under the following accession numbers: GSE214965 (DRUG-Seq; multiplexed RNA-Seq of 20 drugs, 2 time points), GSE214966 (scRNA-Seq; 4 patients at baseline), GSE214967 (scRNA-Seq; patient sample after Vortioxetine vs DMSO treatment), and GSE214968 (RNA-Seq; Vortioxetine time course). Previously published single-cell RNA-Seq datasets analyzed in this study are publicly available at GEO under accession numbers GSE117891 and GSE131928. The publicly available GRCh38 human reference genome was used to align RNA-Seq reads. Proteomics and phosphoproteomics data can be accessed via Panorama (https://panoramaweb.org/GlioB.url). DIA and phosphopeptide enrichment datasets are available from MASSIVE (ftp://massive.ucsd.edu/v04/MSV000090357/). Drug-target annotations and protein-protein interaction data were retrieved from the following publicly available databases: Drug Target Commons (DTC; https://drugtargetcommons.fimm.fi/) and STRING (https://string-db.org/). Other publicly available databases used in this study include DAVID (https://david.ncifcrf.gov/), KEGG (https://www.genome.jp/kegg/), Gene Ontology (http://geneontology.org/), and PathwayNet (http://pathwaynet.princeton.edu/). Data provided via Supplementary Tables include ex vivo drug response of glioblastoma cells (pharmacoscopy scores; Supplementary Table 2), transcriptome-wide neural- and patient-specificity scores derived from three scRNA-Seq datasets (Supplementary Table 3), and in silico COSTAR drug screening results across 1,120,823 compounds (Supplementary Data 2). Source data corresponding to each figure is provided with the manuscript.

Research involving human participants, their data, or biological material

Policy information about studies with human participants or human data. See also policy information about sex, gender (identity/presentation), and sexual orientation and race, ethnicity and racism.

Reporting on sex and gender	Our glioblastoma cohort (n=62 patients; including both prospective and retrospective cohorts and validation patient samples) comprises of 29 females and 33 males identified by their sex. This results in a sex ratio of 46.8% female to 53.2% male present in our cohort. The cohort represents a random sampling of the disease population, where surgical samples were collected from enrolled patients without any exclusion criteria other than the pathological diagnosis being IDH-wildtype glioblastoma. Gender information was not collected as it was not relevant to the current study.
Reporting on race, ethnicity, or other socially relevant groupings	Information on race, ethnicity, or other socially relevant groupings was not collected for the purpose of this study.
Population characteristics	Population characteristics including sex, age, MGMT promoter methylation status, and frequent genetic alterations are reported as a metadata table in Supplementary Table 1.
Recruitment	The glioblastoma cohort represents a random sampling of the disease population, where surgical samples were collected from enrolled patients without any exclusion criteria other than the pathological diagnosis being IDH-wildtype glioblastoma. For the retrospective bio-banked tissue, samples were further selected based on quality control measures including cell viability, cell number, and the amount of debris present in the sample. There was no limit on tumor size for the human samples included in the study and no selection bias of the enrolled patients.
Ethics oversight	Surgically removed tumors were collected at the University Hospital of Zurich (Universitätsspital Zürich, USZ) with approval by the Institutional Review Board, ethical approval number KEK-StV-Nr.19/08, BASEC number 2019-02027 and 2021-00652.

Note that full information on the approval of the study protocol must also be provided in the manuscript.

Field-specific reporting

Please select the one below that is the best fit for your research. If you are not sure, read the appropriate sections before making your selection.

☒ Life sciences ☐ Behavioural & social sciences ☐ Ecological, evolutionary & environmental sciences

For a reference copy of the document with all sections, see nature.com/documents/nr-reporting-summary-flat.pdf

Life sciences study design

All studies must disclose on these points even when the disclosure is negative.

Sample size	For the prospective glioblastoma patient cohort, no sample size determination was performed a priori as the effect size and variability of ex vivo drug response among patients was unknown prior to the study. We processed patient samples according to their surgical availability between August 2019 - December 2021. Our sample sizes build upon other successful glioblastoma studies investigating patient explants such as: https://doi.org/10.1016/j.cell.2019.11.036.
Data exclusions	No data was excluded from the study
Replication	Top neuroactive drugs identified in this study was validated across patient ex vivo samples (n=27 patients), glioblastoma cell lines (n=4 cell lines), patient-derived cultures (n=3 lines), and mouse in vivo models (n=5 trials). For high-content image-based drug screening, drug plates had the following number of replicate wells per drug/concentration. Glioblastoma drug plate; drug, n=3 wells; DMSO, n=16 wells. Neuroactive drug plate; drug, n=4 wells; DMSO, n=16-24 wells; Oncology drug plate; drug, n=4 wells; DMSO, n=16 wells. For other biological measurements such as siRNA-mediated gene silencing, DRUG-Seq, RNA-Seq, and proteomic profiling, a minimum of 3-4 technical or biological replicates across different time points were measured. In vivo mouse experiments were repeated across n=5 trials. DRUG-Seq was performed across two independent sequencing experiments, while scRNA-Seq analysis was validated across n=3 independent datasets. All attempts at experimental replication were successful and reported in the study.
Randomization	As this was a prospective observational study conducted with patient surgical tissue, randomization of patient samples was not necessary as patients were not allocated into different groups. For high-content image-based assays (e.g. drug screening, siRNA screening), to control for plate effects due to laser illumination, drugs or other biological/chemical agents were dispensed into the 384-well plates using an Echo 550

liquid handler (Labcyte) at their respective concentrations in a randomized plate layout. For mouse in vivo experiments, mice were randomly allocated into the different treatment arms.

Blinding

Blinding was not relevant to our study as their was no intervention in the study design.

Reporting for specific materials, systems and methods

We require information from authors about some types of materials, experimental systems and methods used in many studies. Here, indicate whether each material, system or method listed is relevant to your study. If you are not sure if a list item applies to your research, read the appropriate section before selecting a response.

Materials & experimental systems

n/a	Involved in the study
☐	☒ Antibodies
☐	☒ Eukaryotic cell lines
☒	☐ Palaeontology and archaeology
☐	☒ Animals and other organisms
☒	☐ Clinical data
☒	☐ Dual use research of concern
☒	☐ Plants

Methods

n/a	Involved in the study
☒	☐ ChIP-seq
☐	☒ Flow cytometry
☐	☐ MRI-based neuroimaging

Antibodies

Antibodies used

Alexa Fluor® 488 anti-S100 beta (1:1000, Abcam, #ab196442, clone EP1576Y), PE anti-NESTIN (1:150, Biolegend, #656806, clone 10C2), Alexa Fluor® 488 anti-CD3 (1:300, Biolegend, #300415, clone UCHT1), Alexa Fluor® 647 anti-CD45 (1:300, Biolegend, #368538, clone 2D1), Alexa Fluor® 488 anti-NESTIN (1:150, Biolegend, #656812, clone 10C2), Alexa Fluor® 555 anti-S100 beta (1:1000, Abcam, #ab274881, clone EP1576Y), PE anti-CD3 (1:300, Biolegend, #300441, clone UCHT1), Alexa Fluor® 647 anti-Tubulin Beta 3 (1:1000, Biolegend, #657406, clone AA10), Alexa Fluor® 555 anti-Cleaved Caspase-3 (1:500, Cell Signaling Technology, #9604S), Alexa Fluor® 546 anti-HOMER (1:300, Santa Cruz Biotechnology, #sc-17842 AF546, clone D-3), PE anti-CFOS (1:300, Cell Signaling Technology, #14609S, clone 9F6), FITC anti-ATF4 (1:300, Abcam, #ab225332), Alexa Fluor® 488 anti-JUND (1:300, Santa Cruz Biotechnology, #sc-271938 AF488, clone D-9), Alexa Fluor® 594 anti-CD45 (1:300, Biolegend, #368520, clone 2D1), Alexa Fluor® 488 anti-Vimentin (1:500, Biolegend, #677809, clone O91D3), anti-Connexin43 (1:500, Cell Signaling Technology, #83649T), anti-EGFR (1:300, Abcam, #ab98133), anti-CHI3L1 (1:300, Cell Signaling Technology, #47066S, clone E2L1M), anti-Nestin (1:150, Biolegend, #656802, clone 10C2), anti-S100 beta antibody (1:300, Abcam, #ab215989, clone EP1576Y), anti-Ki67 (1:300, Cell Signaling Technology, #9129S, clone D3B5), donkey anti-sheep IgG (H+L) cross-adsorbed secondary antibody, Alexa Fluor™ 488 (1:500, Thermo Scientific, #A11015), goat anti-mouse IgG (H+L) highly cross-adsorbed secondary antibody, Alexa Fluor™ Plus 555 (1:500, Thermo Scientific, #A32727), goat anti-rabbit IgG (H+L) highly cross-adsorbed secondary antibody, Alexa Fluor Plus 647 (1:500, Thermo Scientific, #A32733)

Validation

All primary antibodies used in the study had confirmed species reactivity against human antigens according to the manufacturer's website. All primary antibodies used in the study are commercially available and validation data for their respective application are noted on the manufacturer's website.

Eukaryotic cell lines

Policy information about cell lines and Sex and Gender in Research

Cell line source(s)

LN-229 (ATCC, #CRL-2611) and LN-308 were cultured in Dulbecco's modified Eagle medium (DMEM, #41966, Gibco) supplemented with 10% fetal bovine serum (FBS, #10270106, Gibco). ZH-161 and ZH-562 was generated from freshly isolated tumor tissue and cultured in Neurobasal medium (NB, #21103049, Gibco). LN-229 is a cell line derived from a female patient while LN-308, ZH-161, and ZH-562 are cell cultures derived from male patients.

Authentication

Cell lines (LN-308, ZH-161, and ZH-562) were authenticated at the Leibniz Institute DSMZ (Braunschweig, Germany) by short tandem repeats (STR) analysis of DNA that involves matching mutation profiles between the original tissue and derived cell line. LN-229 was not authenticated as it was bought directly from the vendor (ATCC).

Mycoplasma contamination

Cell lines were not tested for mycoplasma contamination.

Commonly misidentified lines
(See ICLAC register)

No commonly misidentified cell lines were used in the study.

Animals and other research organisms

Policy information about studies involving animals; ARRIVE guidelines recommended for reporting animal research, and Sex and Gender in Research

Laboratory animals

CD1 female nu/nu mice (Janvier, Le Genest-Saint-Isle, France) of 6 to 12 weeks of age

Wild animals	No wild animals were used in the study.
Reporting on sex	Female mice were used in this study.
Field-collected samples	No field collected samples were used in the study.
Ethics oversight	All animal experiments were done under the guidelines of the Swiss federal law on animal protection and were approved by the cantonal veterinary office (ZH98/2018)

Note that full information on the approval of the study protocol must also be provided in the manuscript.

Plants

Seed stocks	*Report on the source of all seed stocks or other plant material used. If applicable, state the seed stock centre and catalogue number. If plant specimens were collected from the field, describe the collection location, date and sampling procedures.*
Novel plant genotypes	*Describe the methods by which all novel plant genotypes were produced. This includes those generated by transgenic approaches, gene editing, chemical/radiation-based mutagenesis and hybridization. For transgenic lines, describe the transformation method, the number of independent lines analyzed and the generation upon which experiments were performed. For gene-edited lines, describe the editor used, the endogenous sequence targeted for editing, the targeting guide RNA sequence (if applicable) and how the editor was applied.*
Authentication	*Describe any authentication procedures for each seed stock used or novel genotype generated. Describe any experiments used to assess the effect of a mutation and, where applicable, how potential secondary effects (e.g. second site T-DNA insertions, mosiacism, off-target gene editing) were examined.*

Flow Cytometry

Plots

Confirm that:

☒ The axis labels state the marker and fluorochrome used (e.g. CD4-FITC).

☒ The axis scales are clearly visible. Include numbers along axes only for bottom left plot of group (a 'group' is an analysis of identical markers).

☒ All plots are contour plots with outliers or pseudocolor plots.

☒ A numerical value for number of cells or percentage (with statistics) is provided.

Methodology

Sample preparation	Cryopreserved single-cell suspensions of glioblastoma patients samples were thawed in reduced serum media (DMEM containing 2% FBS) and used for subsequent flow cytometry experiments. Single-cell suspensions of patient samples were prepared by dissociating surgically derived tumor tissue with Collagenase IV (1mg/ml) and DNaseI (0.1mg/ml) using the gentle MACS Octo Dissociator.
Instrument	BD FACSAriaTM Fusion Cell Sorter
Software	FlowJo 10.4.2
Cell population abundance	Post-sort samples were used for subsequent single-cell RNA-sequencing experiments. Abundances of relevant cell populations such as glioblastoma cells, immune cells, and other tumor microenvironment cell types were determined based on their single-cell transcriptomes and marker gene expression outlined in Extended Data Figure 1e,f.
Gating strategy	FACS gates were set based on CD45 (Alexa Fluor® 594 anti-CD45, 1:20, Biolegend, #368520, clone 2D1), SYTOX Blue and DRAQ5 intensities to isolate live CD45+ and CD45- populations separately as shown in Extended Data Figure 1b. FSC-A and SSC-A gates were first set to only exclude small objects that did not resemble cells, then a doublet-exclusion gate was set on FSC-W and FSC-H. A live cell gate was set to select DRAQ5+SYTOX- viable cells. Finally, gating on CD45 distinguished between immune cells and non-immune cells and were sorted separately. The two populations were mixed back at different ratios to enrich for non-immune cells prior to single-cell RNA-sequencing.

☒ Tick this box to confirm that a figure exemplifying the gating strategy is provided in the Supplementary Information.

Magnetic resonance imaging

Experimental design

Design type	*Indicate task or resting state; event-related or block design.*
Design specifications	*Specify the number of blocks, trials or experimental units per session and/or subject, and specify the length of each trial*

Design specifications	*or block (if trials are blocked) and interval between trials.*
Behavioral performance measures	*State number and/or type of variables recorded (e.g. correct button press, response time) and what statistics were used to establish that the subjects were performing the task as expected (e.g. mean, range, and/or standard deviation across subjects).*

Acquisition

Imaging type(s)	*Specify: functional, structural, diffusion, perfusion.*
Field strength	*Specify in Tesla*
Sequence & imaging parameters	*Specify the pulse sequence type (gradient echo, spin echo, etc.), imaging type (EPI, spiral, etc.), field of view, matrix size, slice thickness, orientation and TE/TR/flip angle.*
Area of acquisition	*State whether a whole brain scan was used OR define the area of acquisition, describing how the region was determined.*

Diffusion MRI ☐ Used ☐ Not used

Preprocessing

Preprocessing software	*Provide detail on software version and revision number and on specific parameters (model/functions, brain extraction, segmentation, smoothing kernel size, etc.).*
Normalization	*If data were normalized/standardized, describe the approach(es): specify linear or non-linear and define image types used for transformation OR indicate that data were not normalized and explain rationale for lack of normalization.*
Normalization template	*Describe the template used for normalization/transformation, specifying subject space or group standardized space (e.g. original Talairach, MNI305, ICBM152) OR indicate that the data were not normalized.*
Noise and artifact removal	*Describe your procedure(s) for artifact and structured noise removal, specifying motion parameters, tissue signals and physiological signals (heart rate, respiration).*
Volume censoring	*Define your software and/or method and criteria for volume censoring, and state the extent of such censoring.*

Statistical modeling & inference

Model type and settings	*Specify type (mass univariate, multivariate, RSA, predictive, etc.) and describe essential details of the model at the first and second levels (e.g. fixed, random or mixed effects; drift or auto-correlation).*
Effect(s) tested	*Define precise effect in terms of the task or stimulus conditions instead of psychological concepts and indicate whether ANOVA or factorial designs were used.*

Specify type of analysis: ☐ Whole brain ☐ ROI-based ☐ Both

Statistic type for inference (See Eklund et al. 2016)	*Specify voxel-wise or cluster-wise and report all relevant parameters for cluster-wise methods.*
Correction	*Describe the type of correction and how it is obtained for multiple comparisons (e.g. FWE, FDR, permutation or Monte Carlo).*

Models & analysis

n/a	Involved in the study
☐	☐ Functional and/or effective connectivity
☐	☐ Graph analysis
☐	☐ Multivariate modeling or predictive analysis

Functional and/or effective connectivity	*Report the measures of dependence used and the model details (e.g. Pearson correlation, partial correlation, mutual information).*
Graph analysis	*Report the dependent variable and connectivity measure, specifying weighted graph or binarized graph, subject- or group-level, and the global and/or node summaries used (e.g. clustering coefficient, efficiency, etc.).*
Multivariate modeling and predictive analysis	*Specify independent variables, features extraction and dimension reduction, model, training and evaluation metrics.*
